# ACAP1 assembles into an unusual protein lattice for membrane deformation through multiple stages

**DOI:** 10.1371/journal.pcbi.1007081

**Published:** 2019-07-10

**Authors:** Chun Chan, Xiaoyun Pang, Yan Zhang, Tongxin Niu, Shengjiang Yang, Daohui Zhao, Jian Li, Lanyuan Lu, Victor W. Hsu, Jian Zhou, Fei Sun, Jun Fan

**Affiliations:** 1 Department of Materials Science and Engineering, City University of Hong Kong, Hong Kong, China; 2 National Laboratory of Biomacromolecules, CAS Center for excellence in biomacromolecules, Institute of Biophysics, Chinese Academy of Sciences, Beijing, China; 3 School of Chemistry and Chemical Engineering, South China University of Technology, Guangzhou, Guangdong, China; 4 Division of Rheumatology, Immunology and Allergy, Brigham and Women’s Hospital, and Department of Medicine, Harvard Medical School, Boston, Massachusetts, United States of America; 5 School of Biological Sciences, Nanyang Technological University, Singapore; 6 Center for Biological Imaging, Institute of Biophysics, Chinese Academy of Sciences, Beijing, China; 7 City University of Hong Kong Shenzhen Research Institute, Shenzhen, China; 8 Center for Advanced Nuclear Safety and Sustainable Development, City University of Hong Kong, Hong Kong, China; Bogazici University, TURKEY

## Abstract

Studies on the Bin-Amphiphysin-Rvs (BAR) domain have advanced a fundamental understanding of how proteins deform membrane. We previously showed that a BAR domain in tandem with a Pleckstrin Homology (PH domain) underlies the assembly of ACAP1 (Arfgap with Coil-coil, Ankryin repeat, and PH domain I) into an unusual lattice structure that also uncovers a new paradigm for how a BAR protein deforms membrane. Here, we initially pursued computation-based refinement of the ACAP1 lattice to identify its critical protein contacts. Simulation studies then revealed how ACAP1, which dimerizes into a symmetrical structure in solution, is recruited asymmetrically to the membrane through dynamic behavior. We also pursued electron microscopy (EM)-based structural studies, which shed further insight into the dynamic nature of the ACAP1 lattice assembly. As ACAP1 is an unconventional BAR protein, our findings broaden the understanding of the mechanistic spectrum by which proteins assemble into higher-ordered structures to achieve membrane deformation.

## Author summary

Membrane remodeling is needed for a wide range of cellular events. Our current understanding of how proteins bend membrane to achieve such remodeling has come, in large part, from studies on proteins that contain the BAR domain. These studies have elucidated that the BAR domain dimerize to form a crescent-shaped structure. This structure induces membrane deformation through scaffolding and/or insertion of an amphipathic loop into the membrane. Some BAR-containing proteins also contain a PH domain in tandem with their BAR domain. We have found previously that one such protein, known as ACAP1, relies primarily on the PH domain, rather than the BAR domain, for membrane deformation. To explain this phenomenon, we have utilized, in the current study, molecular dynamics simulations, Monte Carlo calculations, and together with Cryo-electron sub-tomography experiments, to gain insights into how the ACAP1^BAR-PH^ protein assembles into an unusual protein lattice through multiple dynamics stages. The results are further verified by functional experiments.

## Introduction

Membrane deformation is needed for a wide range of cellular processes, including intracellular transport, organelle biogenesis, cell division, and cell motility [1,2]. Some of the best characterized proteins that deform membrane possess a Bin-Amphiphysin-Rvs (BAR) domain [3–8]. Studies on how this domain structure induces membrane curvature have suggested two mechanisms. One way involves scaffolding [5,9,10]. The dimerization of the BAR domain produces a curved, banana-like structure, which can then impose curvature onto the underlying membrane through electrostatic interactions. A second way involves protein insertion into the membrane [4,11,12]. Some BAR domains possess an amphipathic helix, which can insert into one leaflet of the membrane to create asymmetry between the bilayers, resulting in curvature induction. In recent years, a more detailed understanding of how BAR proteins induce membrane curvature has come from high-resolution electron microscopy (EM) studies, which couples cryo-EM with protein crystallography, resulting in an atomic-level view of how BAR proteins are organized into higher-ordered structures on membrane for curvature induction [5,8,11,13,14]. Emerging from these studies has been a general paradigm for how BAR proteins deform membrane. Briefly, the dimeric BAR structure acts as the basic repeating unit, which propagates along its length through tip-to-tip interactions, and also laterally through side-to-side interactions, to assemble into lattice structures that appear as “criss-crossing” strands on membrane for curvature induction [15,16]. A number of recent work has revealed how BAR domains [17–19] or banana-shaped rods [20] cluster to form scaffolds on membranes. While the lattice assembly was shown to be dynamical in nature, the intermediary stages of the assembly process have been less clear.

We have recently uncovered a different paradigm for how a BAR protein deforms membrane. A subset of BAR proteins possesses the BAR domain in tandem with a Pleckstrin Homology (PH) domain. Early studies found that the PH domain in these proteins is critical for membrane deformation, but the explanation had remained elusive [21–24]. We recently addressed this puzzle in studying a GTPase-activating protein (GAP) for ADP-Ribosylation Factor 6 (ARF6), known as ACAP1 (Arfgap with Coil-coil, Ankryin repeat, PH domain 1). Besides its traditional role as a regulator of the ARF6, ACAP1 also acts as an ARF6 effector [25]. This involves ACAP1 functioning as a coat protein, which deforms endosomal membrane to generate transport carriers for recycling to the plasma membrane [26–28]. To achieve a better understanding of how ACAP1 acts in this capacity, we recently pursued structural studies on the BAR-PH tandem of ACAP1 (ACAP1^BAR-PH^), which is the minimal portion of ACAP1 sufficient for membrane deformation. The solved structure suggested that, rather than the BAR domain engaging the membrane, the PH domain contacts the membrane. We then pursued functional studies to show that a loop in the PH domain likely inserts into the membrane to impart curvature. Thus, rather than its BAR domain, the main driver of membrane deformation for ACAP1 is its PH domain [8].

We also pursued high-resolution EM-based studies to gain insight into how ACAP1^BAR-PH^ organizes into a higher-order lattice structure to achieve membrane deformation. The result revealed another key difference between how ACAP1 versus how a conventional BAR protein deforms membrane. Whereas conventional BAR domains contact the membrane along the entire length of their curved structure, through the concave side [29,30], only one end of the ACAP1^BAR-PH^ dimer contacts the membrane. Consequently, whereas the basic repeating unit of protein lattices formed by conventional BAR proteins is the dimer, the tetramer constitutes the basic repeating unit in the ACAP1 lattice, which is achieved by the end of an ACAP1^BAR-PH^ dimer that does not contact the membrane interacting instead with the mid-portion (arch) of another dimer, resulting in an “end-to-arch” interaction between two dimers in forming a tetramer [8]. Here, we have pursued multiple complementary approaches to gain insight into how ACAP1 assembles into this unusual lattice structure for membrane deformation.

## Results

### Major contact regions within the ACAP1^BAR-PH^ protein lattice

**Fig 1:**
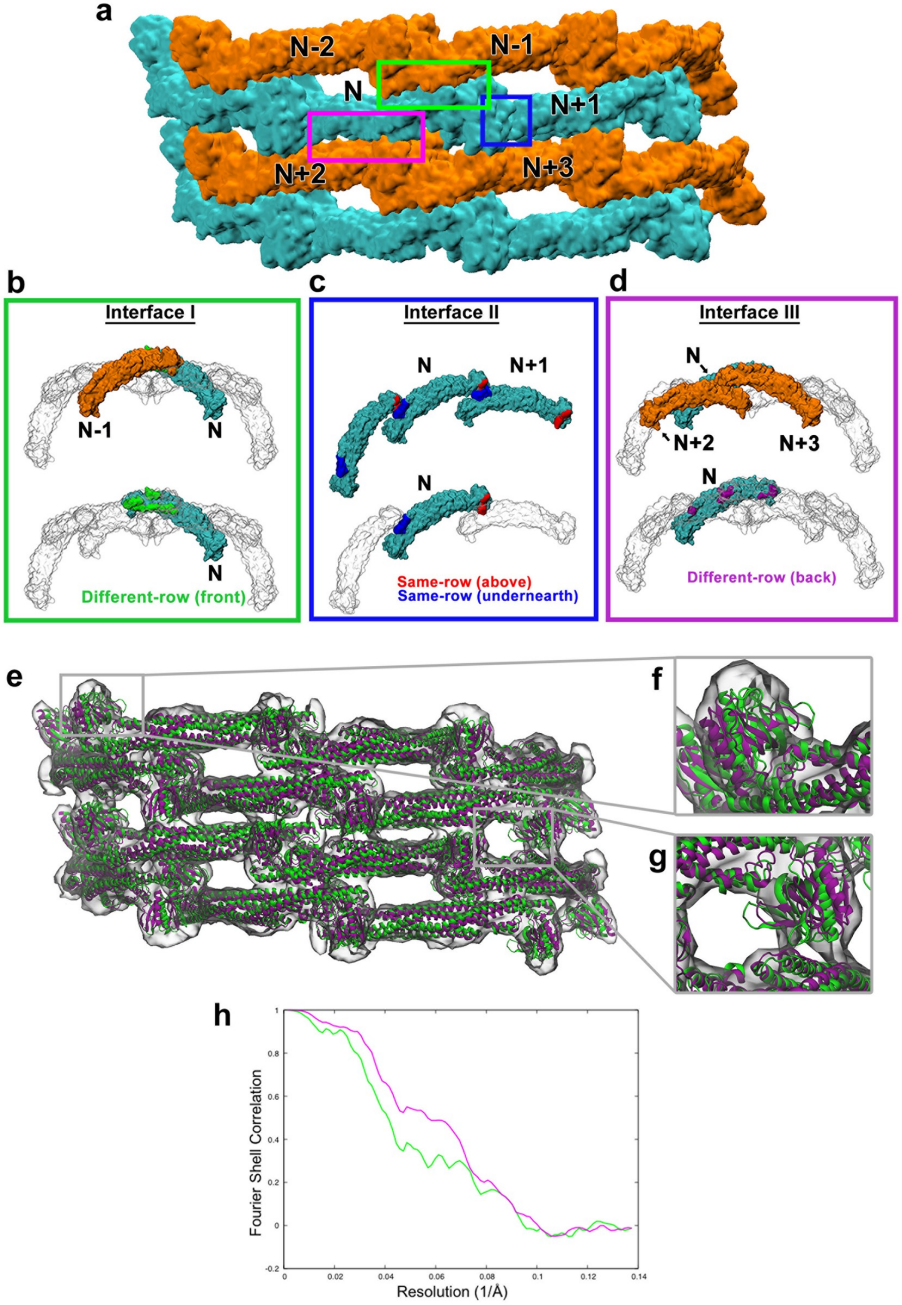
Configuration of a partial ACAP1^BAR-PH^ protein lattice. (**a**) Surface diagram of the ACAP1^BAR-PH^ protein lattice in the class 1 MDFF simulation with dimers labelled. Three interaction interfaces are enclosed with three boxes of colours: green for the front interactions (Interface I), blue for same-row interactions (Interface II), and magenta for the back interactions (Interface III). (**b-d**) Surface diagram of ACAP1^BAR-PH^ dimers shown in cyan, orange or transparent in the upper panels. Cyan and orange colours indicate dimers in different rows. In the lower panels, some dimers were made transparent to clearly show contact residues for each interacting region. For Interface I, the contact residues on dimer N are shown in green in **b.** The surface of other dimers in the lattice are colored in grey. For Interface II, the contact residues from the PH domain are shown in red, and those from the BAR domain are shown in blue in **c**. The contact residues for the back interaction are shown in purple in **d**. (**e**) The density map includes four rows of proteins and superposition of ACAP1^BAR-PH^ protein structures before (green) and after (purple) MDFF simulations. (**f-g**) Enlarged views of the improved protein structures. **(h)** Comparison of Fourier shell correlation (FSC) curves. The green curve is the FSC curve between the rigid body fitting model and the EM map, while the purple one is the curve between the refined model and the EM map.

We had previously reconstructed an ACAP1^BAR-PH^ protein lattice on tubulated portions of liposomes, which predicts the structural organization needed for membrane deformation [8]. Overall, the ACAP1^BAR-PH^ dimer is predicted to oligomerize, both longitudinally and laterally, in forming helical strands that wrap around a tubular membrane with regular periodicity, resulting in an “criss-crossing” appearance by which ACAP1 coats the membrane (Fig 1a). Initially, performing closer inspection of this arrangement, we could appreciate three major regions of protein contacts (highlighted by green, blue, and magenta boxes in Fig 1a). The relative positions of these three interfaces can be further defined when considering a grouping of six contiguous ACAP1^BAR-PH^ dimers within the lattice structure, which consist of two dimers in the upper helical strand (defined as positions N-2 and N-1), two dimers in the middle strand (defined as positions N and N+1), and two dimers in the lower strand (defined as positions N+2 and N+3) (Fig 1a).

Interface I (green box in Fig 1a) is an inter-strand interaction, and involves the two dimers of the upper strand (positions N-2 and N-1) contacting the two dimers of the middle strand (positions N and N+1). This interface is further highlighted in Fig 1b, in which the relative position of one dimer in the upper strand (N-1, colored orange) is shown in relation to one dimer in the middle strand (N, colored cyan). Interface II (blue box in Fig 1a) is an intra-strand interaction, and involves two adjacent dimers in the middle strand interacting with each other (positions N and N+1). This interaction is further highlighted in Fig 1c, with one dimer (position N, colored in cyan) interacting with the other dimer (position N+1, also colored in cyan). We had previously noted the unusual nature of this contact, as it involves an “end-to-arch” interaction between the PH domain (at the end) of one dimer with the BAR domain (at the mid-section) of the other dimer in generating a tetramer, which is also the basic repeating unit of the lattice structure [8]. Interface III (magenta box in Fig 1a) is another inter-strand interaction, and involves two dimers in the middle strand (positions N and N+1) interacting with two dimers in the lower strand (positions N+2 and N+3). This interaction is further highlighted in Fig 1d, in which the relative position of a dimer in the middle strand (position N, colored cyan) to that of two dimers in the lower strand (positions N+2 and N+3, colored in orange) is shown.

Our previous reconstruction of the ACAP1^BAR-PH^ lattice did not achieve sufficient resolution to identify specific residues that mediate these three major interfaces of contacts. Thus, we initially sought to refine the resolution by pursuing a type of molecular dynamics (MD) simulation, known as MD flexible fitting (MDFF). This is a computational approach that employs MD simulations to fit atomic structures into cryo-EM density maps [31] and has been successfully applied to improving the structural details of multiple macromolecular assemblies [32–35]. A grouping of 12 contiguous ACAP1^BAR-PH^ protein dimers were embedded onto the cryo-EM density map (summarized in S1 Fig; map resolution 14 Å). This resulted in ~140,000 protein atoms being analyzed, or ~2,340,000 total atoms when the solvent and ions were also included. The crystal structure of ACAP1^**BAR-PH**^ protein (PDB: 4NSW) was used for the fitting.

Protein configurations before (colored in green) and after (colored in magenta) simulations are shown in Fig 1e, with further details of typical refined regions shown in Fig 1f and 1g. The Fourier Shell Correlation (FSC) profile between the map and the model validated the improvement of the structure after simulation (Fig 1h). The result also revealed that a number of interacting residues would be missed by the rigid-body docking method (S2 Fig), and many of these residues are predicted to reside in the three major interfaces (S3 Fig and S1 Table).

**Fig 2:**
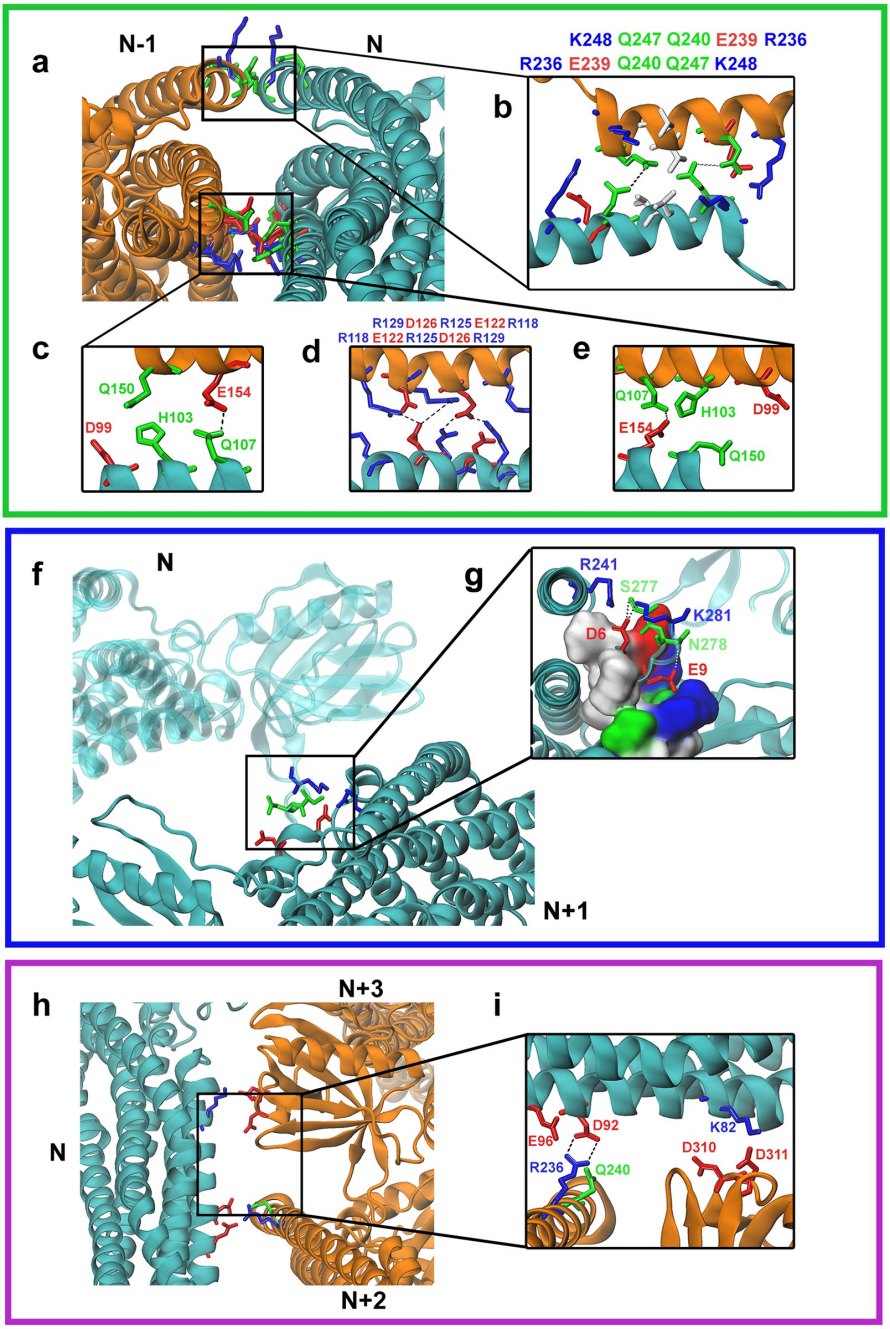
Identification of key interacting residue pairs between protein dimers inside the ACAP1^BAR-PH^ protein lattice. (**a**, **f**, **h**) Enlarged views of the interacting regions. Residues are coloured according to residue types, i.e., negative: red; Positive: blue; Polar: green; Non-polar: white. (**b**) A symmetrically anti-parallel interacting residue pairs at Interface I. (**c-e**) Another symmetrically anti-parallel interacting residue pairs at Interface I. (**g**) A binding pocket from dimer N+1, shown in surface representation, forms Interface II by interacting with residues from dimer N. (**i**) Interacting residues at Interface III. In close-up panels, dotted lines indicate salt bridges or hydrogen bonds between charged residues.

In the more refined structure, interface I is predicted to be composed of two contacting regions (Fig 2a), with the upper of these two contacts highlighted in Fig 2b, and the lower contact highlighted in Fig 2c, 2d, and 2e. For the upper contact, multiple residues (R236, E239, Q240, Q247 and K248) in the α4 helix of the BAR domain in the dimer N are predicted to interact with the same group of residues in the dimer N-1. This interaction occurs symmetrically and in an anti-parallel fashion, which would be analogous to two persons shaking their right hands. The simulation results further predicted that residue Q240 forms H-bonds with Q247 and K248 in this contact. For the lower contact, simulation results predicted that multiple residues (D99, H103, Q107, R118, D122, R125, D126, R129, Q150 and E154) in the α2 helix of the BAR domain in the dimer N would interact symmetrically and in an anti-parallel fashion to the same group of residues in the dimer N-1. This contact could be further sub-divided into three regions, denoted as left (Fig 2c), central (Fig 2d) and right (Fig 2e). The central region has multiple charged residues (R118, E122, R125, D126, R129), with the dimer N interacting symmetrically and anti-parallel with the dimer N-1 (Fig 2c), while the left (Fig 2d) and right (Fig 2e) regions have several polar residues. Overall, Interface I is created by four salt-bridges, residue pairs E122-R125, and E122-R129, twice in each dimer pair, and six H-bonds, residues Q107-E154, E122-R125, E122-R129, twice in each dimer pair due to anti-parallel symmetry (summarized in Table 1).

**Table 1:** Salt bridges/H-bonds between ACAP1^BAR-PH^ dimers inside the protein lattice.

Subunit A	Residue 1	Subunit B	Residue 2	Occupancy (%)*
Salt Bridges**				
**Interface I**				
N or N-1	E122	N-1 or N	R125	94.50
	E122		R129	99.58
**Interface II**				
N	D6	N+1	K281	72.50
**Interface III**				
N	D92	N+2	R236	73.25
H-Bonds**				
**Interface I**				
N or N-1	Q107	N-1 or N	E154	44.67
	E122		R125	89.50
	E122		R129	85.67
	Q240		Q247	43.92
	Q240		K248	15.75
**Interface II**				
N	D6	N+1	K281	65.88
	D6		S277	37.75
	E9		N278	20.25
	R241		S277	10.33
**Interface III**				
N	D92	N+2	R236	62.75

*The occupancy of a salt bridge or H-bond is defined as the percentage of time it has been observed throughout the equilibrated MDFF trajectory.

**Any salt bridge or H-bond formed between residues with occupancy greater than 10% are shown.

Interface II is predicted to be created by a portion of the BAR domain in the dimer N+1 forming a binding pocket, and a portion of the PH domain in the dimer N forming a loop that inserts into the binding pocket (Fig 2f). Specifically, a portion of the α0 helix (residues 1 to 20) of the BAR domain in the dimer N+1 is predicted to form a binding pocket, while residues 276 to 282 of the PH domain in the dimer N is predicted to form the insertion loop (designated as Loop1 and located between the β1 and β2 sheets of the PH domain). The refined model also suggested some specific interactions. These include salt bridges and H-bonds formed by residue D6 of the BAR domain in the dimer N+1 interacting with residue K281 of the PH domain in the dimer N. H-bonds are also formed between residues D6 of the BAR domain (in the dimer N+1) and S277 of the PH domain (in the dimer N), and between residues E9 of the BAR domain and N278 of the PH domain. In addition, the insertion loop (Loop1 in the PH domain of the dimer N) is found to interact with part of the α4 helix (residues 234 to 245) of the BAR domain in the dimer N+1. This interaction should further stabilize the main contact described above, created by the insertion of Loop1 in the dimer N into the binding pocket formed by the α0 helix in the dimer N+1 (Fig 2f). An H-bond was also observed between R241 of the BAR domain in the dimer N and S277 of the PH domain in the dimer N+1 (Fig 2g and Table 1).

Interface III is predicted to be generated by two α helices in the BAR domain of the dimer N interacting with the BAR domain in the dimer N+2 and the PH domain in the dimer N+3 (Fig 2h). Specifically, D92 of α2 helix in the BAR domain of the dimer N forms a salt bridge and H-bond with R236 in the α4 helix of the BAR domain in the dimer N+2 (Fig 2i and S1 Table). Another part of the α2 helix (charged residue K82) in the dimer N contacts with the β4/β5 loop of the PH domain (charged residues D310 and D311) in the dimer N+3 (Fig 2i).

Overall, as the electrostatic interactions are about one order of magnitude higher than the van der Waals interactions, additional analysis suggested that charged residues at the three interfaces should provide the main driving force for the protein contacts (S1 Table).

### Functional confirmation of the simulation predictions

**Fig 3:**
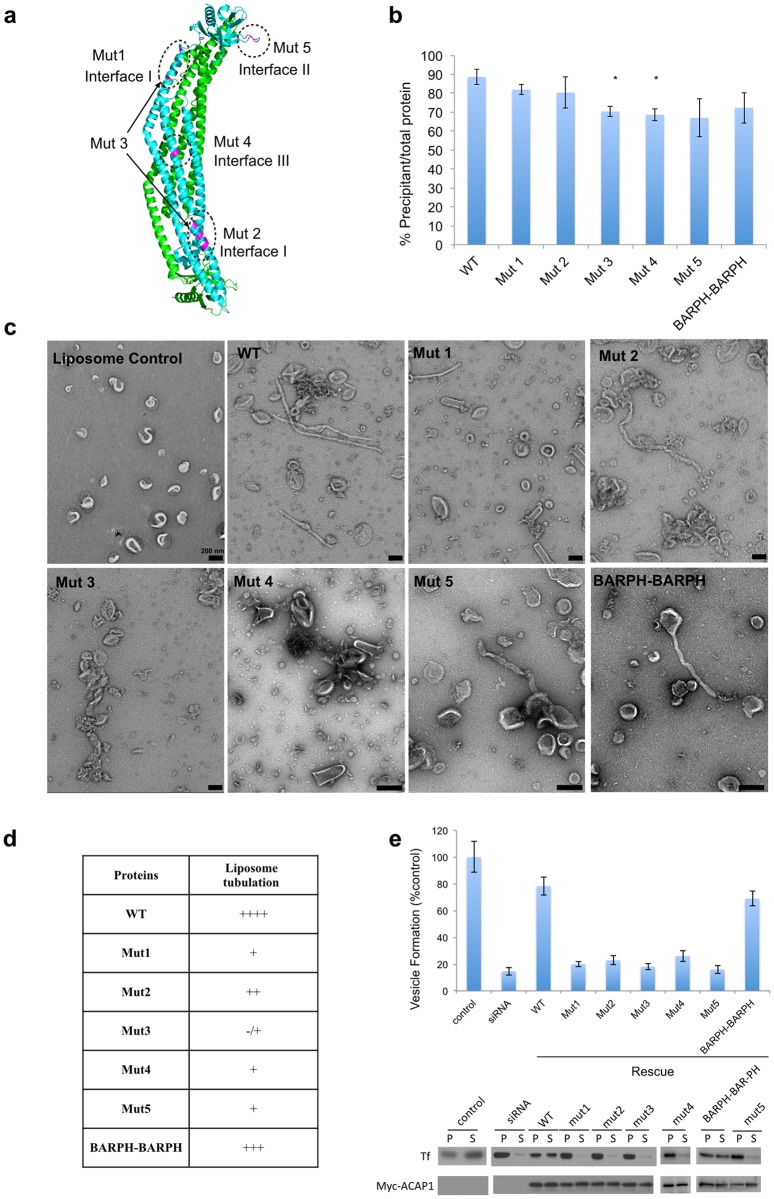
Mutagenesis studies to assess membrane binding and tabulation *in vitro* and endocytic recycling *in vivo*. (**a**) Location of mutations indicated on the structure of ACAP1^BAR-PH^ (PDB: 4NSW) are coloured in magenta. Interfaces implicated in higher-order assembly of ACAP1^BAR-PH^ are circled with dotted lines. Mutations are indicated: Mut 1, R236A/E239A/K248A; Mut 2, R118A/E122A/R125A/D126A; Mut 3, a compound mutation of Mut 1 and 2, R118A/E122A/R125A/D126A/R236A/E239A/K248A; Mut 4, D92A/R236A; Mut 5, BARPH-Deletion, S277-N278-F280-K281. (**b**) Binding of mutant forms of ACAP1^BAR-PH^ (as indicated) to liposomes is assessed by co-precipitation assay and quantified from three independent experiments. All error bars represent the SD from three independent experiments. The degree of significance involves comparison between wild-type and different mutants, with *p < 0.01. (**c**) Negative-stain EM visualizing liposomes incubated either with or without ACAP1^BAR-PH^ and its mutants (as indicated). Mutations are the same as in (**a**), the scale bar represents 200nm as indicated. (**d**) The relative tubulation ability of indicated protein is expressed as ++++ (very strong), +++ (strong), ++ (moderate), + (weak), or—/+ (almost nothing). (**e)** The reconstitution of recycling vesicles from the endosomal membrane was performed by incubating the siACAP1-treated HeLa cell total membrane and siACAP1-treated HeLa cytosol, with the addition of cytosols containing over-expressed ACAP1 mutants. The level of vesicle formation after the incubation was then quantified. All error bars represent the SE from three independent experiments.

We next sought to confirm the above predictions through functional studies. For interface I, we generated three sets of mutations. One set targeted a cluster of clustered charged residues, R236, E239 and K248, which were predicted to participate in the upper contact in Interface I (see Fig 2b). Mutation of these residues to alanines (R236A/E239A/K248A) resulted in mutant 1 (Mut1) (Fig 3a). A second set of mutations targeted another set of charged residues R118, E122, R125 and D126, which were predicted to participate in the lower contact in Interface I (see Fig 2d). Mutation of these residues to alanines (R118A/E122A/R125A/D126A) resulted in mutant 2 (Mut2) (Fig 3a). We also generated mutant 3 (mut3), which combined the mutations in Mut1 and Mut2 (R118A/E122A/R125A/D126A/R236A/E239A/K248A) (Fig 3a). For Interface III, we targeted a predicted critical interaction between the D92 residue in the BAR domain of one dimer and the R236 residue in the BAR domain of another dimer (see Fig 2i). Mutation of these residues to alanines (D92A/R236A) generated mutant 4 (Mut4) (Fig 3a).

Interface II was more challenging to disrupt. This contact involves two ACAP1^BAR-PH^ dimers interacting through an “end-to-arch” interaction in forming a tetramer (see Fig 1c). Thus, disruption of this interaction required that we only target one of the two PH domains in the ACAP1^BAR-PH^ dimer. We had previously overcome this hurdle by noting that ACAP1^BAR-PH^ dimerizes in a symmetrical and anti-parallel fashion, and this orientation could be preserved by covalently linking two ACAP1^BAR-PH^ monomers in a “head-to-tail” fashion [8]. Importantly, the resulting fusion protein (referred as BARPH-BARPH) was shown to be functional, retaining the ability to tubulate liposomes similar to that seen for wild-type ACAP1^BAR-PH^ (which dimerizes through non-covalent interaction) [8]. Thus, to target Interface II, we generated a dimer fusion protein and then mutated residues S277, N278, F280, and K281 in only one of the two PH domains in this fusion protein, resulting in mutant 5 (Mut5) (Fig 3a).

We then pursued functional studies. Initially, we examined membrane binding by ACAP1, and found that all five mutations impaired the recruitment of ACAP1 to membrane to some extent (Fig 3b). Subsequently, we pursued additional approaches to assess membrane deformation by ACAP1. First, we examined the ability of ACAP1 to induce liposome tubulation, as done previously [8], and found that all mutations affected this ability of ACAP1 (Fig 3c). We also pursued a second assay. ACAP1 acts as a coat protein in generating transport carriers for endocytic recycling, and we had previously established the reconstitution of ACAP1-dependent carrier formation from endosomal membrane [8]. Performing this reconstitution, we confirmed that all five mutants also reduced the ability of ACAP1 to support vesicle formation from endosomal membrane (Fig 3e). We also performed MD simulations of Mut3, Mut4 and Mut5, which revealed disassociation of dimers from tetramer state, leading to disruption to the protein lattice (S4 Fig). Thus, these results confirmed in complementary ways that the MDFF simulations had identified key protein-protein contact sites that enable dimeric ACAP1 to assemble into a higher-ordered lattice structure for membrane deformation.

### Asymmetrical binding as the energetically favourable state

We then considered a potential clue. Whereas the mutations had modest effects on the membrane-binding assay, they exhibited more severe effects in the two assays of membrane deformation, i) liposome tubulation and ii) carrier formation from endosomal membrane. This disparity suggested that the initial stage of lattice assembly, which involves the recruitment of ACAP1 to membrane, may be dynamic, and thus could not be captured completely by a simple membrane-binding assay. Thus, to explore this possibility, we next embarked on simulation studies, which are better suited in capturing dynamic situations.

We initially pursued an algorithm based on parallel tempering Monte Carlo (PTMC) simulation [36], which investigates the orientation by which the ACAP1^BAR-PH^ dimer is adsorbed onto a negatively charged membrane surface under different surface charge densities (SCD) and ionic strengths (IS). Simulation parameters (SCD and IS) are listed in S2 Table. The parameters for modelling the membrane surface have been described previously [37]. Moreover, a coarse-grained united-residue model was employed, i.e., each amino acid of the protein was reduced to an interaction site centered at the α-carbon of the residue, with parameters as previously described [37]. Since the inter-molecular interactions between the charged surface and the protein are important, and the intra-molecular interactions of the protein itself are less important, the protein structure was kept rigid. This modelling approach has been successfully applied previously to elucidate the adsorption orientation of lysozyme [36] and antibodies [37] on charged surfaces.

**Fig 4:**
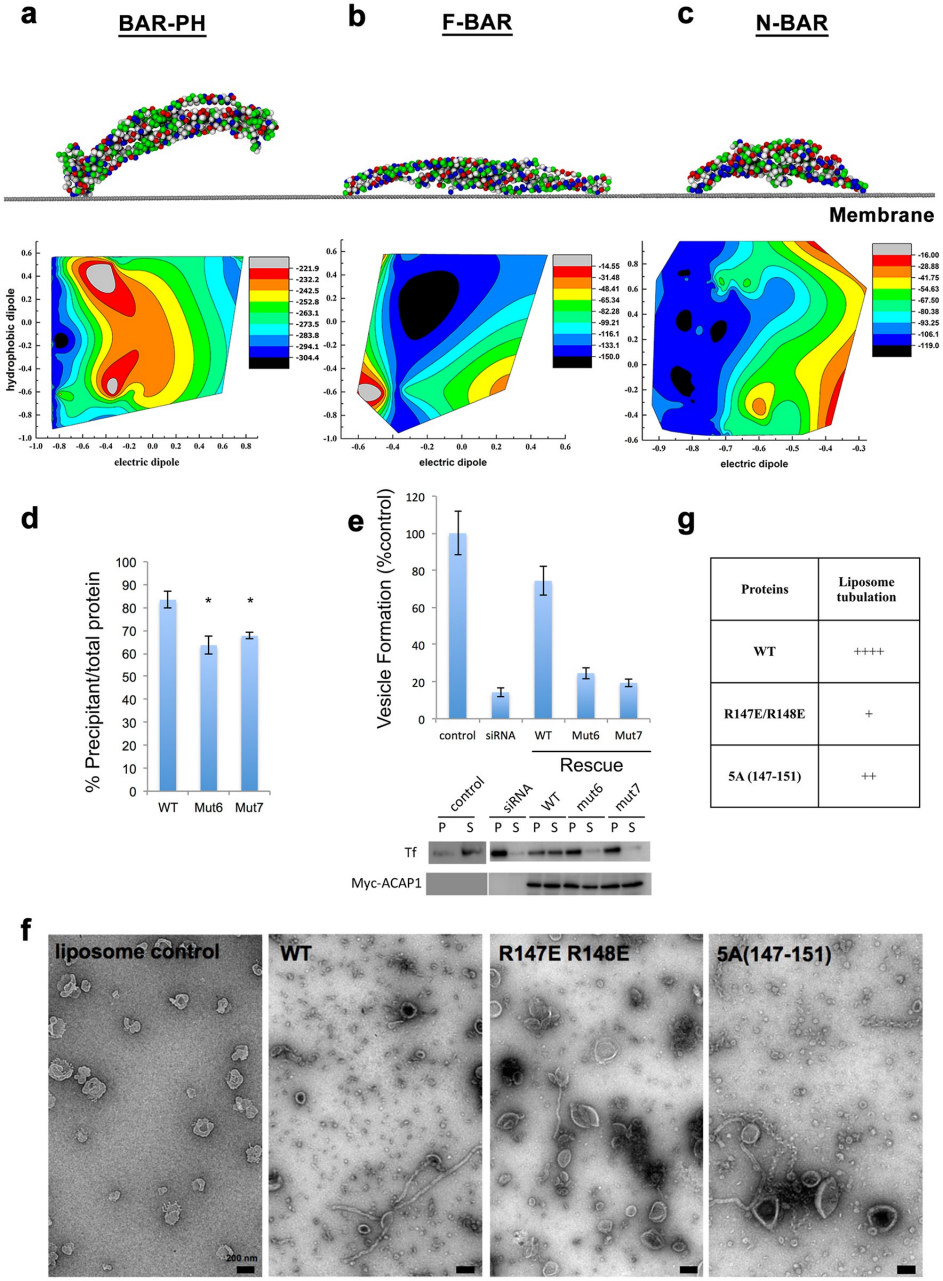
Binding configurations revealed by PTMC simulations. **(a-c)** PTMC results of (**a**) BAR-PH, (**b**) F-BAR, and (**c**) N-BAR. The energetically minimized system configurations were shown, in which the coarse-grained beads were coloured according to the type of the residue. The modelled membranes were represented by grey flat surfaces. Samplings of two predefined dipoles (electric and hydrophobic) during calculation were shown in contour maps. **(d)** The binding of mutant forms of ACAP1^BAR-PH^ (as indicated) to liposomes is assessed by co precipitation assay and quantified. All error bars represent the SD from three independent experiments. The degree of significance involves comparison between wild-type and different mutants, with *p < 0.01. Mutations are indicated: Mut 6, R147E/R148E; Mut 7, R147A/R148A/A149/Q150A/Q151A. **(e)** The reconstitution of recycling vesicles from the endosomal membrane was performed as Fig 3e. The level of vesicle formation after the incubation was then quantified. All error bars represent the SD from three independent experiments. **(f)** Negative-stain EM visualizing liposomes incubated either with or without ACAP1^BAR-PH^ and its mutants (as indicated), the scale bar represents 200 nm as indicated. **(g)** The relative tubulation ability of indicated protein is expressed as Fig 3d.

For the ACAP1^BAR-PH^ dimer (PDB ID: 5H3D), which was predicted to be neutrally charged, there was no significant electrostatic repulsion with the negatively charge surface. When IS and SCD were set as 0.18 M and -0.127 C·m^-2^, respectively, “one-end-on” orientation (shown in Fig 4a) became favored, with the potential energy (-303.5 kJ/mol), which is lower than that of symmetrically binding (-274.4kJ/mol). We also manually constructed a symmetric dimer structure, and similarly the asymmetric binding model is more favorable. These findings predicted that the ACAP1^BAR-PH^ dimer would be approaching the membrane asymmetrically, and thereby explaining why its final configuration in the lattice structure shows only one of the two ends in the dimer contacting the membrane.

We sought to confirm this prediction in two ways. First, we sought validation that PTMC simulation would accurately predict the recruitment behavior of BAR proteins that have been well characterized. For an F-BAR protein (PDB ID: 2EFK [38]) (Fig 4b), which has -6e net charges, the electrostatic repulsion still existed, due to the relatively uniform distribution of charges on protein surface and a smaller dipole moment. In this case, adsorption strength was relatively weak. At a low surface charge density, electrostatic interactions were similar to van der Waals interactions. At a high surface charge density, electrostatic interactions became dominant, and this was caused by the shielding effect of solution ionic strength on the surface charge. We obtained almost the same optimal orientation with SCD of 0.007 and 0.127, which was a “lying-on-side” orientation that involves the entire length of the protein interacting with the model surface. Notably, this mode of membrane interaction has been predicted previously as an intermediate stage of lattice assembly for this BAR protein [5]. We further noted that the key residues predicted to mediate membrane binding include K56, E92, K104, Q107, K114, K122, R125, Q160, A167, Q170, and K174 (S3 Table), and a number of these residues have already been confirmed by previous functional studies [5,10,38].

For the N-BAR protein (PDB ID: 1X03 [12]) (Fig 4c), which has -20e net charge, there was significant electrostatic repulsion with negatively charge surface, and consequently adsorption strength was significantly reduced. However, due to the uneven distribution of charged residues on the protein surface and the shielding effect of the strong solution ionic strength on the electrostatic repulsion, the negatively charged protein still adsorbed onto the negatively charge surface. With increasing surface charge density, adsorption became stronger. This resulted in almost the same optimal orientation with SCD = 0.007 and SCD = -0.127. In these cases, the “two-end-on” orientation was the optimal orientation. Moreover, as shown in S3 Table, the key adsorption residues were predicted as R174, Q175, G176, K177, I178, and E182, with the positively charged R174 and K177 interacting electrostatically with the negatively charged surface being predicted to be the main driving force. Notably, these predictions have also been confirmed previously by functional studies [4,12].

We next pursued a second way of validating the results of the PTMC simulations on ACAP1. Besides predicting an asymmetric approach to the membrane by ACAP1, PTMC simulations also predicted specific residues that are critical for this behaviour. These residues could be sub-divided into two regions of ACAP1, with one cluster (F280, K281, D322, and E325) located in the PH domain, and another cluster (R147, R148, A149, Q150, and Q151) located in the BAR domain. A critical role for the clustered residues in the PH domain was expected, as our previous structural elucidation of the ACAP1 lattice on membrane revealed that this region provides the sole means by which the lattice contacts the underlying membrane [8]. We had also performed functional studies that confirmed this situation [8]. In contrast, a role for the clustered residues in the BAR domain was unexpected, as this region was not observed to contact the membrane in the previously elucidated structure of the ACAP1 lattice on membrane [8]. Thus, we next pursued functional studies to confirm this unexpected finding.

We generated two types of mutations. When the R147 and R148 residues were mutated to glutamates (R147E, and R148E; Mut 6; Fig 4d), we found that membrane binding of the ACAP1^BAR-PH^ protein was reduced to some extent (Fig 4e). When the entire cluster was converted to alanines (R147A, R148A, A149, Q150A, and Q151A; Mut 7; Fig 4d), membrane binding was affected similarly (Fig 4e). In the liposome tubulation assay, we found that the mutations reduced membrane deformation by ACAP1 more dramatically (Fig 4f). Similarly, we found that that mutations had a more dramatic effect in reducing the reconstitution of ACAP1-dependent endocytic recycling carrier formation (Fig 4g).

We further noted that these results on mutants 6 and 7 paralleled those seen above for the effects of mutants 1–5. That is, the membrane-binding assay was only modestly affected by the mutations, while the assays of membrane deformation, liposome tubulation and carrier formation from endosomal membrane, were affected more drastically. Moreover, as the residues in the BAR domain were not seen to contact the membrane in the ACAP1 lattice structure that we had previously elucidated [8], the collective considerations suggested that the BAR domain residues likely participated in ACAP1 contacting the membrane in a dynamic manner.

The position of the residues in the BAR domain also suggested how this dynamic recruitment could occur. In the solved structure of the ACAP1 dimer, these residues are located in close proximity to the residues in the PH domain, which we had previously documented to participate in membrane contact [8]. Moreover, when considering that the residues in the BAR domain are positioned more laterally than these residues in the PH domain, we concluded that the participation of the BAR domain residues would result in the ACAP1 dimer initially contacting the membrane in a more “tilted” manner than that would have been predicted if only the residues in the PH domain were involved (S4 Fig).

### The ACAP1^BAR-PH^ dimer shows intrinsically asymmetric dynamics

Unlike other BAR proteins, such as PICK1 [39], which form tetramer or octamer in the solution, ACAP1 exists as a dimer at the concentration from 10 to 50 μM [8]. We next addressed a fundamental question arising from the prediction that the ACAP1 dimer would initially contact the membrane through only one of its two ends. As this dimer is structurally symmetrical, how can it behave asymmetrically for membrane contact? To gain further insight, we next pursued additional simulation studies.

**Fig 5:**
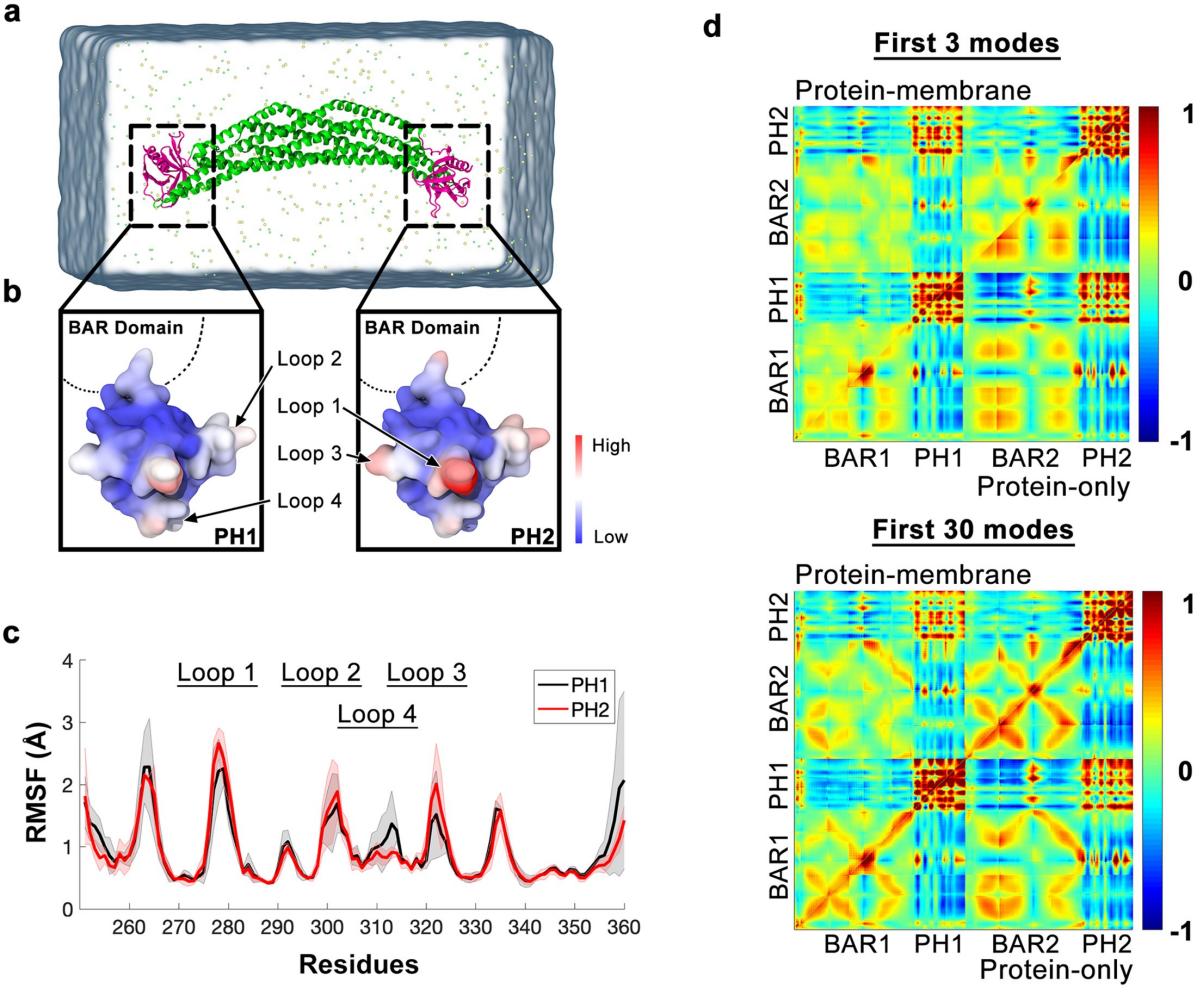
The asymmetric insertion of the ACAP1^BAR-PH^ dimer occurs during initial membrane recruitment. **(a)** Simulation set-up of one ACAP1^BAR-PH^ dimer in solvent with ions. The BAR domain is coloured in green while the PH domain is coloured in magenta. Ionic particles are represented in yellow and light green bubbles. **(b)** Projection of residue B-factors onto the molecular surface of the PH domains. Magnitudes were represented from low to high with the colour blue to red. Important contacting loops were marked with arrows. **(c)** RMSF profile of the two PH domains of ACAP1^BAR-PH^ dimer were coloured in black and red. Errors (shown in shadows) were calculated from three individual simulations, each using a 200-ns trajectory. **(d)** The correlated fluctuations of the Cα atoms in the ACAP1^BAR-PH^ dimer by integrating the first 3 or 30 principle components calculated from MD trajectories.

Initially, we performed multiple independent MD simulations of a single ACAP1^BAR-PH^ dimer in solution (Fig 5a). By examining the fluctuation of C_α_ atoms in the protein backbone, we uncovered that the ACAP1^BAR-PH^ dimer was intrinsically asymmetric in its dynamics. B-factors computed from atomic displacement of the residues indicated the relatively active regions on the molecular surface of the PH domain (Fig 5b). Remarkably, residue loops in the PH domain, which facilitated the interaction between the ACAP1^BAR-PH^ dimer and the membrane surface were also found to be dynamic in simulations, even when no membrane was present. The two PH domains of the protein dimer showed different but consistent root-mean-square-fluctuation (RMSF) profiles in all three independent simulations (Fig 5c). One of the PH domains (PH1) always had relatively larger fluctuations in Loop 1 (residue 276–282) (>2.5 Å) than the other PH domain (PH2). Also, the RMSF of Loop 4 (residue 322–235) in one PH domain, serving as the linking residues between Loop 2 and Loop 3, was higher than that in the other PH domain. These fluctuations were predicted to disrupt membrane binding by the PH domain on one end of the ACAP1^BAR-PH^ dimer greater than that of the PH domain in the other end. Note that the initial protein structure of MD simulations is the crystal structure 4NSW, which consists of two chemically identical chains. In our MD simulations, either PH domain may possess a higher RMSD. This phenomenon may come from an allosteric effect [40]. The asymmetry of the RMSF are consistent with the “one-end-on” configuration, i.e., it would be much easier for PH2 to bind to the membrane than PH1.

Principle component analysis (PCA) of the MD trajectories further supported the above conclusion. The two monomers within the ACAP1^BAR-PH^ dimer were found to exert different but correlated dynamics even without the presence of the membrane (Fig 5d). In the first few low-frequency modes (sorted by the associated eigenvalues in descending order), introduction of a model membrane surface altered the residue fluctuation correlation matrix for both monomers. Generally, the two monomers had a stronger dynamic correlation without the lipid molecules. Integrating more normal modes ceased the difference between proteins, regardless of the presence of a model membrane. Projection of the trajectories onto the first two essential dynamics of the protein also revealed two distinct dynamical states (S7 Fig). Further analysis employing the time-lagged independent component analysis (TICA) [41,42], which finds a subspace with maximized autocorrelation, also show that the two dynamical states are distinguishable and independent of minor revision to molecular force-field (S8 Fig). Thus, these findings suggested that the distal regions of the ACAP1 dimer exert unique internal dynamics even in solution, and this behavior diminishes after it comes toward the membrane.

### Cryo-EM studies uncover further dynamics in the assembly of the ACAP1 lattice

**Fig 6:**
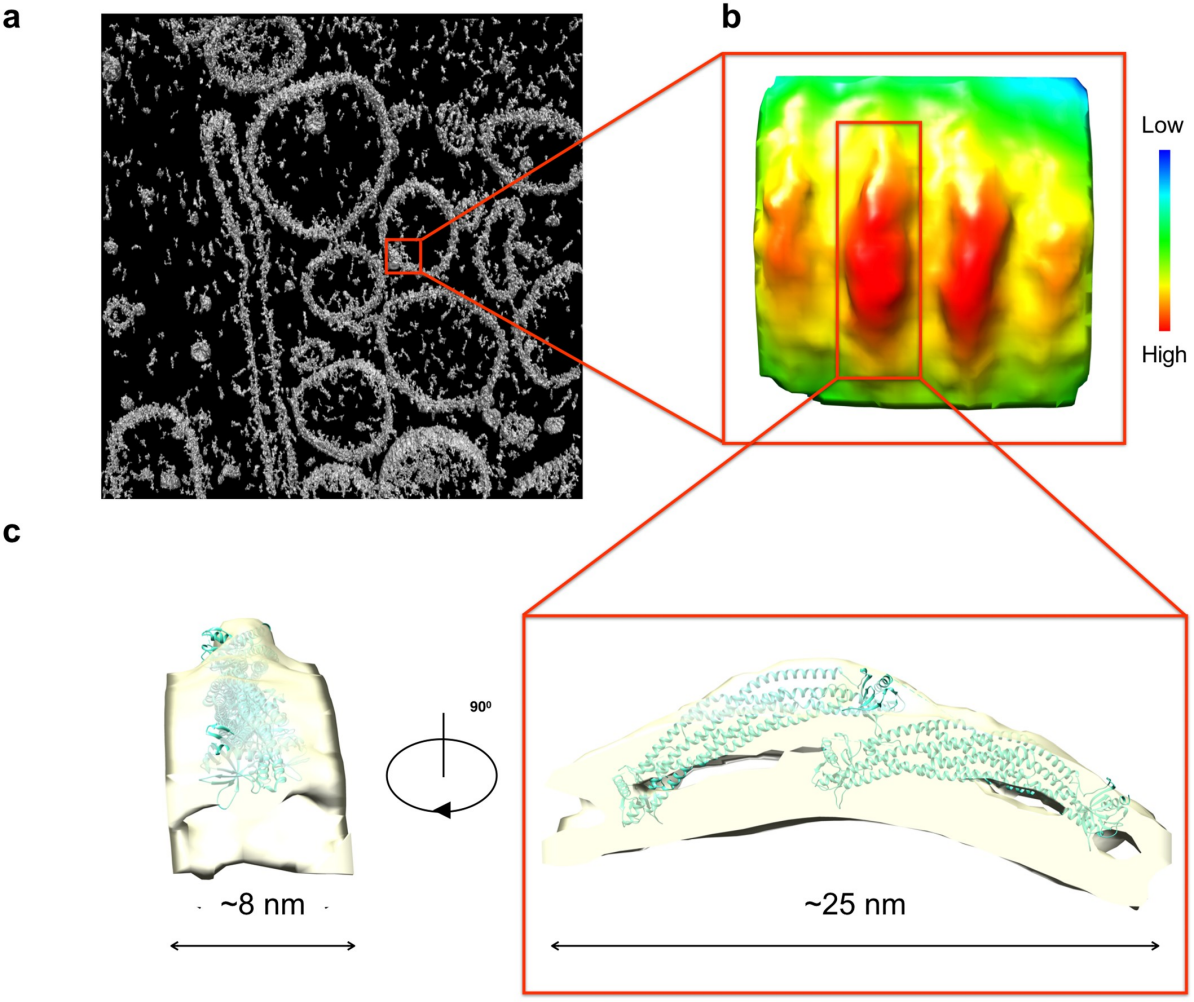
CryoEM reconstruction and sub-tomogram averaging of ACAP1^BAR-PH^ on liposomes in situ. **(a)** Cryo-electron tomograms section showing coat layer arranged around tubular and spherical membranes. **(b)** Average of 233 coat particles picked out from tomogram in top view and side view, showing the ACAP1^BAR-PH^ tetramer and neighbouring particles. Rainbow colours represent different density values. **(c)** Cross-section and side view of the central density at different threshold values (grey mesh, high; transparent orange, low), fitted by structure of ACAP1^BAR-PH^ (PDB: 4CKG) with colour of cyan.

We then pursued another line of investigation that further reinforced the dynamic nature by which ACAP1 assembles into its protein lattice structure. Previous studies on the assembly of BAR proteins into lattice structures on membrane have sought insight into intermediary stages by examining the coating organization of these proteins on non-tubulated portion of liposomes, which is predicted to reveal a stage of lattice assembly toward its final functional form [5]. Taking a similar strategy, we initially also observed ACAP1^BAR-PH^ to coat liposomes on both non-tubulated and tubulated regions (Fig 6a). Thus, to gain insight into an intermediary stage of ACAP1 lattice assembly, we next sought to deduce how it is organized on the non-tubulated portions of liposomes.

Sub-tomogram averaging suggested that ACAP1 on these liposomes formed curved densities, which are organized into particle units that are ~25 nm long and ~8 nm wide. These particles were flanked by adjacent similarly shaped particles that run roughly parallel (Fig 6b). We fitted a structural model of ACAP1^BAR-PH^ dimer (PDB: 4CKG) onto the curved density map and found that the coated layer is longer than the length (~16 nm) of the ACAP1^BAR-PH^ dimer. Thus, each particle unit was predicted to have more than one dimer. Further analysis revealed that the EM map could match closely with two ACAP1^BAR-PH^ dimer molecules packed with each other via the interactions between the PH domain and BAR domain of the adjacent protein dimer in the same row (Fig 6c). Notably, these tetramers exhibit a more elongated shape as compared to tetramers that we had previously observed on tubulated liposomes.

Collectively, the above findings suggested that the ACAP1^BAR-PH^ dimer packs onto planar membrane (non-tubulated portions of liposomes) with a different arrangement than that previously seen on curved membrane (tubulated portions of liposomes). Thus, we concluded that we have detected an intermediary stage of ACAP1 lattice assembly on membrane. In this case, it involves tetramers of ACAP1 having already been formed on membrane, but not having reached its final configuration seen on tubulated portions of liposomes. Importantly, such a result further reinforced the dynamic nature of the ACAP1 lattice assembly.

## Discussion

We have pursued multiple, and complementary, approaches to gain insights into how ACAP1 assembles into an unusual lattice structure for membrane deformation. The unusual organization was originally revealed by pursuing high-resolution, structural, and EM-based studies [8]. However, due to the limited resolution previously achieved, we were unable to address a key question. What are the specific protein-protein contacts that mediate this unusual lattice organization?

In the current study, we initially pursued MDFF simulations to refine the model, which predicted specific residues to participate in forming the key contact points. The nature of the residues involved suggested that electrostatic interactions would be the main driver in assembly of the ACAP1^BAR-PH^ lattice structure. Further notable was that a number of the interacting residues predicted by MDFF simulations would have been missed by the rigid-body docking method, and importantly many of these residues are predicted to participate in the major contact points within the lattice.

We then pursued functional studies to confirm the predicted key contact points. One assay examined ACAP1 binding to membrane, while two other assays assessed membrane deformation by ACAP1, these being liposome tubulation and carrier formation from endosomal membrane. Although all three assays were affected by mutations of the predicted residues, a notable difference was that the mutations affected the ability of ACAP1 to deform membrane more severely than its ability to bind membrane. Thus, when also considering that the two assays of membrane deformation are predicted to interrogate a more complex situation than the simpler membrane-binding assay, we concluded that ACAP1 is likely recruited to the membrane more dynamically than that could be detected by the membrane-binding assay, which monitored a more static situation.

We then pursued simulation studies, as they are better suited for dynamic situations. ACAP1 exists in solution as a dimer that forms a symmetrical curved structure. However, unlike the conventional BAR proteins, which have been predicted to be recruited to the membrane symmetrically, having both ends of their dimeric curved structure contacting the membrane, PTMC simulations predicted that the ACAP1 dimeric structure would be recruited to the membrane asymmetrically, having only one of the two ends of the curved structure contacting the membrane. This prediction also suggested a key puzzle to address. How can a symmetrical structure bind to the membrane in an asymmetric fashion? Pursuing further simulation studies, our results predicted that the PH domain in the ACAP1 dimer exhibits dynamic fluctuations in solution, which would explain why the ACAP1 dimer initially contacts the membrane asymmetrically.

We had previously pursued a simpler simulation approach, which allowed us to model only how the concave side of the curved ACAP1 dimer approaches the membrane in a perpendicularly fashion. In the current study, pursuing a more comprehensive analysis, which queries all angles by which ACAP1 approaches the membrane, we have achieved a remarkable insight. The simpler simulation that we had previously pursued identified key residues in the PH domain to be critical for membrane binding. This was expected, as the previous structural elucidation of the ACAP1 lattice also shows these residues to be involved in membrane binding[8]. In contrast, the more comprehensive simulation analyses that we have pursued in the current study predict that a region in the BAR domain would also participate in ACAP1 contacting the membrane to initiate lattice assembly. This was unexpected, as this region of ACAP1 had not been observed to contact the membrane in our previous structural elucidation of the ACAP1 lattice.

Importantly, the location of these BAR domain residues also suggests how they could be participating in membrane contact in a dynamic manner. Relative to the PH domain that contacts the membrane, the predicted residues in the BAR domain is located more proximal and lateral. Thus, the participation of these residues in membrane binding predicts that the curved dimeric ACAP1 structure would contact the membrane in a more “tilted” fashion. Notably, such a scenario is reminiscent of how a conventional BAR protein has been predicted to undergo assembly to form a functional protein lattice on membrane, as its curved dimer has also been proposed to contact the membrane initially through its side rather than perpendicularly through its concave surface. Thus, despite exhibiting notable differences in their final lattice organization on membrane, with the dimeric ACAP1 exhibiting asymmetric contact with the membrane and the conventional BAR domain exhibiting symmetric contact with the membrane, both types of lattices are predicted to transit through an intermediary stage of assembly that involve the more lateral surface of their curved dimeric structure coming into contact with the membrane.

We also pursued another line of investigation that further reinforces the dynamic nature by which ACAP1 assembles into a lattice structure for membrane deformation. We had previously pursued structural and EM-based studies to elucidate a lattice structure formed by ACAP1 on tubulated liposomes, which is predicted to be the protein organization that deforms membrane. In the current study, we have further combined structural and EM-based studies with computational approaches to uncover how ACAP1 is organized on the non-tubulated portion of liposomes, which is predicted to gain insight into an intermediary stage of lattice assembly. As cryo-electron tomography and sub-tomogram averaging suggested a stage of lattice assembly in which tetramers of ACAP1 have been formed but have not reached their final configuration seen on tubulated liposomes, we conclude that the dynamic assembly of the ACAP1 lattice extends even to the point when tetramers have been formed. Thus, ACAP1 exhibits dynamic behavior not only in its initial stage of recruitment to membrane, but also subsequently during its assembly on membrane into a higher-ordered structure for membrane deformation.

## Methods

### Protein preparation

ACAP1^BAR-PH^ (a.a. 1–377) and different mutants were cloned into the plasmid pGEX-6P-1 (GE Healthcare), expressed as GST-tag fusion proteins in *Escherichia coli* BL21 (DE3) cells. Cells were grown in Terrific Broth medium at 37°C until the OD at 600 nm reached 1.2~1.5, and then induced at 16°C for 16 ~18 hours with 0.2 mM IPTG. Cells were harvested and re-suspended in PBS buffer (140 mM NaCl, 2.7 mM KCl, 10 mM Na2HPO4, 1.8 mM KH2PO4, pH 7.4) and lysed by sonication. After centrifugation for 30 minutes at 15,000 rpm, the supernatant was collected and incubated with glutathione-Sepharose 4B at 4°C, and then was washed by PBS buffer. After cleavage using Precision Protease (GE Healthcare) to remove the GST tag, the eluted target proteins were changed to buffer A (50 mM Hepes, pH 7.4, 50 mM NaCl) and stored at -80°C. Site-directed mutations of select residues were performed by overlap PCR. To introduce mutations to only one subunit of dimeric BAR-PH, one copy of BAR-PH was inserted into plasmid pGEX-6P-1 with nonstop, and then another copy with Deletion (S277-N278-F280-K281) was cloned into pGEX-6P-1-BAR-PH, to generate a tandem fusion version Mut5 (BAR-PH-Deletion), with a sequence (GGGSGGRLGSSNSG) as a linker between BAR-PH and deletion.

### Liposome production and sedimentation assay

All lipids were purchased from Avanti Polar lipids. Lipid mixtures were similar to that previously described [8], containing 40% phosphatidylcholine (DOPC), 30% phosphatidylethanolamine (DOPE), 20% phosphatidylserine and 10% of L-α-phosphatidylinositol-4,5-bisphosphate (PI(4,5)P2). They were dried under gas nitrogen and then kept under vacuum for at least three hours. Dry lipid mixtures were suspended in 50 mM HEPES, pH 7.4, 50 mM NaCl for 30 minutes at 37 °C, frozen in liquid nitrogen, and thawed at 37 °C for 5 cycles, and extruded through membrane filters of 0.2 μm for the production of 200 nm liposomes. For the sedimentation assay, the 200 nm liposomes (1 mg/ml) and ACAP1^BAR-PH^ proteins (0.2 mg/ml) were incubated for 60 minutes at room temperature before ultra-centrifugation at 250,000 g for 15 minutes. The supernatants and pellets were then subjected to SDS-PAGE analysis.

### Reconstitution of recycling vesicles from endosomal membrane

The reconstitution system was performed essentially as previously described [8]. Briefly, to collect total membranes and cytosol, HeLa cells were incubated with biotin-conjugated transferrin (Tf) at 4°C for 1 hour and then at 37°C for 15 minutes, which allows a pool of Tf receptor (TfR) at the cell surface to accumulate at the early endosome in tracking endosomal membranes. Cells were then resuspended in buffer (20mM HEPES, pH 7.4, 150 mM NaCl), followed by homogenization by passing through a 23-gauge needle 16 times on ice. The homogenate was then subjected to centrifugation to obtain total membranes (with early endosomes labeled by biotin-conjugated Tf) and cytosol.

To reconstitute recycling vesicles from endosomal membrane, total membranes and cytosol, collected as described above, were incubated with 1mM GTP at room temperature for 30 minutes. To detect the level of recycling vesicles formed after this incubation, the sample was subjected to centrifugation at 13,000 x g for 20 minutes at 4°C to derive pellet fraction (P), which contains organellar membranes, and supernatant fraction, which containing vesicles and cytosol (S). Recycling vesicles were detected in the supernatant fraction by blotting for biotin-conjugated Tf using a horseradish peroxidase-conjugated streptavidin.

To assess the effect of different mutations of ACAP1 on the formation of recycling vesicles, HeLa cells were treated with siRNA against ACAP1, followed by the collection of total membranes and cytosol, as described above. Cytosol was also collected from HeLa cells that overexpressed different mutant forms of myc-tagged ACAP1. Cytosol from the two sources of cells (treated with siRNA against ACAP1 or overexpressing different mutant ACAP1) were mixed at 9:1 ratio, respectively, to obtain physiologic level of different mutant ACAP1 expressed in the cytosol. This resulting cytosol was then incubated with total membrane derived from HeLa cells treated with siRNA against ACAP1. The level of recycling vesicles generated after this incubation was then assessed through the tracking of biotin-conjugated Tf, as described above.

### Molecular dynamics flexible fitting (MDFF)

Simulations employed cryo-EM density maps from one of two classes of protein lattices associated with tubular membranes of various radius. The system contained ~140,000 protein atoms and ~2,340,000 total atoms, including solvent. Six protein tetramers served as the basic configuration unit for MDFF simulations. Initial structures of the protein were based on the crystal structure of ACAP1^**BAR-PH**^ protein (PDB: 4NSW). Proteins were solvated in a box of TIP3P water molecules [43] with 180 mM KCl using VMD [44]. Periodic boundary conditions were introduced to the system.

The MDFF simulations were performed using NAMD 2.11 [45] with CHARMM36 force field [46]. A timestep of 1 fs has been used. Cut-off distance was 10 Å for the non-bonded interactions. Temperature was maintained at 310 K using a Langevin thermostat coupled to all heavy atoms with a damping coefficient of 5 ps^-1^. Restraints for secondary structures were introduced to the system. Symmetrical restraints [47] were also introduced for simulating part of a helical structure.

A total of 27 ns of trajectory was generated. Only data after reaching equilibrium were taken for further analysis. As a result, the final 15 ns of trajectories were used for analysis unless otherwise specified. All quantities shown are the averaged values over the simulation windows.

A salt bridge was defined as a pair of acidic oxygen and basic nitrogen atoms separated by less than 4 Å of distance. A hydrogen bond (H-bond) is defined as a pair of polarized oxygen, nitrogen or sulphur atoms separated by less than 3.5 Å of distance and forming an angle less than 30° with the adjacent hydrogen atom. The occupancy of a salt bridge/H-bond is defined as the percentage of time that the salt bridge/H-bond existed throughout the simulations.

### MD (molecular dynamics) simulations

We have also constructed molecular systems, in which a single ACAP1^BAR-PH^ dimer was placed in solvent with ions. Initial structures of the protein were based on the crystal structure of ACAP1^**BAR-PH**^ protein (PDB: 4NSW). The systems were solvated and ionized with TIP3P water molecules [43] and 180 mM KCl ions using VMD [44]. Periodic boundary conditions were introduced. Three independent simulations have been performed.

The MD simulations were performed using NAMD 2.11 package [45]. The molecular force-fields used were either CHARMM36 [46] or CHARMM36m [48]. A timestep of 2 fs has been used. Electrostatic interactions were calculated using the particle mesh Ewald sum method [49] with a cutoff of 12 Å. Before production runs, the system was minimized in energy, heated to 310 K, and pre-equilibrated by step-wisely releasing harmonically restrained protein backbone and water oxygen atoms. Simulations were then continued in the constant NPT ensemble with 310 K and 1 atm. Langevin thermostats with a damping coefficient of 0.5 ps^-1^, and Langevin-piston barostats [50] with a piston period of 2 ps and a damping time of 2 ps were used.

In total five independent 500 ns of trajectories were generated for an ACAP1 dimer in ionic solvent. Only data after reaching equilibrium were taken for further analysis. Unless otherwise specified, the last 200 ns of data were used for analysis. All quantities presented in this article are averaged values over the chosen windows. The RMSF profile of two PH domains were calculated from three of the trajectories (using CHARMM36 force-fields). Convergence of the RMSF profiles have been confirmed by using a moving 50 ns window starting at time = 100, to 500 ns and lack of large deviations along the principle components of motions (S6 Fig).

### PTMC (parallel tempering Monte Carlo) simulation

A coarse-grained united-residue model [37] was employed to explore the preferred orientation of BAR domain proteins on negatively charged surfaces under different SCD and IS. In the model, each amino acid was reduced to an interaction site centred at the α-carbon of the residue.

As the objective of PTMC simulations is to obtain the favourable orientation of BAR proteins on model charged surfaces, and when also considering that the inter-molecular interactions between protein and surface are important, while the intra-molecular interactions within the protein itself are less important, the protein structures were kept rigid. The charged surfaces had both van der Waals’ (VDW) and electrostatic interactions with the protein. To mimic the membrane bilayer, surfaces were assigned net negative charges and the SCD were calculated according to membranes adopted in experiments. The simplified flat model considered the most essential two factors, surface charge density and ionic strength, which could make the flat model provide a reasonable prediction of protein orientation on membrane surfaces.

The parameters for model surfaces were taken from our previous works [37]. Five replicas, each in the canonical ensemble, were simulated in parallel at different temperatures of 310 K, 500 K, 800 K, 1500 K and 2500 K, which ensured sufficient energy overlap between neighbouring replicas to allow for the acceptance of configuration swaps. The swaps were performed every 500 cycles.

The adsorption and preferred orientation of five BAR domain proteins were studied at different IS and SCD. Orientation angle (θ) is used to quantitatively characterize the orientation of adsorbed proteins on surfaces, which is defined as the angle between the unit normal vector to the surface and the unit vector along the dipole of a protein. The cosine value of this angle (cosθ) was calculated for each possible orientation. The orientation and corresponding preferred configuration of each protein on different charged surfaces at different ionic strengths were then derived. The direction of dipole was also calculated. The total potential energy (U_tot_), VDW potential energy (U_VDW_), electrostatic potential energy (U_ele_) and the cosθ of each protein adsorbed on surfaces at different SCD and IS were also calculated.

The Monte Carlo (MC) simulation in each replica was carried out in a box of 30 nm × 30 nm × 30 nm. Initially, the protein was put 10 nm above the surface with a random orientation. During simulations, the protein was translated and rotated around its centre of mass. The displacement of each move was adjusted to ensure an acceptance ratio of 0.5. A total of 15×10^6^ MC cycles were carried out, in which the first 5×10^6^ cycles for equilibrium and another 10×10^6^ cycles for production. Besides, 310K is the target temperature.

### Negative-stain electron microscopy

The ACAP1^BAR-PH^ and mutant proteins (1 mg/ml) were incubated with liposomes (0.5 mg/ml) for 60 minutes and then the mixture was applied onto a glow-discharged carbon-coated EM grid and stained with uranyl acetate. The EM grids were examined with a transmission electron microscope (FEI Tecnai20 or Talos) and the micrographs were recorded with a Gatan UltraScan1000 CCD camera under the nominate magnification of 9,600X or with a FEI Ceta camera under the nominate magnification of 13,500X.

### Cryo-electron tomography and sub-tomogram averaging

The sample preparation for Cryo-electron tomography was the same as previously described[8]. And tomographic data collection was also the same as described before[8]. Tilt images were aligned using Markerauto[51]. Tomograms were reconstructed using weighted back projection (WBP)^37^.To obtain average results, 976 individual particles were manually picked and cut out using IMOD^36^, To avoid any kind of over-fitting, the initial model for sub-volume alignment was generated by averaging all particles in random orientations[52] and refined using RELION 1.4[53]. Then the model was low pass filtered to 60 Å before subjecting into PEET^37^ for further refinement. The final STA procedure was carried out using PEET, following an iterative angular refinement step. To be noted that the parameter flgWedgeWeight in PEET was set to 0 to include the information in the missing wedge. The final searching step of Euler angle is 1°. Cryo-EM maps were displayed, and fitted with atomic models using UCSF Chimera[54].

## Supporting information

S1 Table Key contacting residues in the ACAP1^BAR-PH^ protein lattice. All residue pairs were summarized from class 1 MDFF trajectory then extracted from those dominating the interaction interface in S3 Fig. The interaction energies were calculated using VMD’s NAMD Energy tool[45]. Number of salt bridges/H-bonds was counted and averaged over the simulation trajectory. Abbreviations: Elec., electrostatic; VDW, Van der Waal’s.
(DOCX)

S2 Table PTMC simulation parameters and results for BAR domains. (DOCX)

S3 Table Key interacting residues from BAR domains identified during protein recruitment PTMC simulations. (DOCX)

S4 Table Statistics of residue conservation using ConSurf (*Nucleic acids research* 44. W1 (2016): W344-W350). 500 sequences were used to sample the list of homologues collected from UniProt to the query, among which 152 unique sequences were further used for conservation analysis. The conservation scores were calculated using Bayesian method and presented by conservation level (9—conserved, 1—variable). MSA data is the number of aligned sequences having an amino acid (non-gapped) from the overall number of sequences at each position. Residues that have been mutated in mutagenesis were colored in red.
(DOCX)

S1 Fig Components of the MDFF simulations. Surface diagram of the ACAP1^BAR-PH^ protein lattice in the class 1 MDFF simulation with dimers labelled. Three interaction interfaces are enclosed with three boxes of colours: green for the front interactions (Interface I), blue for same-row interactions (Interface II), and magenta for the back interactions (Interface III).
(TIF)

S2 Fig Residue pairs at the protein interaction interfaces inside the ACAP1 protein lattice. Residue pairs before and after the MDFF refinement were colored in black and red respectively.
(TIF)

S3 Fig All contact residues between proteins inside an ACAP1^BAR-PH^ protein lattice. **(a)** Contact numbers for each residue coloured according to different interacting regions for class 1. Different colours stand for different interacting interfaces, which are consistent with colours shown in Fig 1. An additional colour (red) has been introduced to Interface II and was used to denote residues from the dimer geometrically above the adjacent dimer at the interface. (**b**) Residue contributions in **a** have been projected onto the molecular surface of an ACAP1^BAR-PH^ dimer. The adjacent dimers have been shown in transparent.
(TIF)

S4 Fig Deviation of the adjacent dimer from its refined structure. To compute the root-mean-square deviation (RMSD) of the mutated protein dimer with respect to its refined structure, one of the dimers from an equilibrated mutated tetramer MD trajectory was aligned to its refined structure obtained from the MDFF simulation, which also served as the initial structure for the mutated tetramer MD simulations. Three independent simulations were performed with mutations stated in experiments and shown in different colors.
(TIF)

S5 Fig Snapshots of the recruitment of a BAR-PH dimer onto the membrane from MD and PTMC simulations. (a) Residues on the BAR domain (especially R147 and R148) were highlighted during the recruitment process. Residues R147 and R148 locate at the distal ends of the BAR domain (initially colored in blue). Residues from the BAR domain that were within 4A of a PIP2 molecule will be shown explicitly and colored according to their electrostatic properties (Blue for positive, red for negative, green to polar and white for hydrophobic). PIP2 molecules that were within 8A of the dimer will also be shown explicitly. (b) A slight “lying-down” orientation was observed for the BAR domain in both MD and PTMC simulations. Residues R147 and R148 were shown in blue spheres in the PTMC snapshot, showing a very similar way of contact seen in the MD snapshots.
(TIF)

S6 Fig Principle component analysis of individual PH domains. The whole 500 ns trajectories were used to compute the principle components. Last 200 ns of the trajectories were projected onto the first two principle components. Colorbar represents the simulation time.
(TIF)

S7 Fig Principle component analysis of trajectories consisting of all PH domains from three independent MD simulations. (a) Three independent MD trajectories were projected onto the first two principle components. Trial 1 and 3 have been clearly identified to have two forms of motion along the first principle of component of dynamics in fluctuations (PC1) indicating that the two PH domains underwent different dynamics. Trial 2 was not observed to have such distinct dynamics along PC1, indicating the possibility of interchangeable of the two dynamical states. (b) For the sake of clarity, the (PC1) was represented using arrows on the molecular surface of PH domain shown in Fig 5b. This PC1 has shown that motions in residues of Loop 1, Loop 3 and Loop 4 have contributed to most of the residual fluctuation and distinguish the two dynamics of the PH domain which is in consistent with the RMSF profile in Fig 5c.
(TIF)

S8 Fig TICA analysis of PH domains from five independent MD simulations. (a) A pseudo free energy surface calculated from the sample densities using the first two PCs of PCA. (b) A pseudo free energy surface of the sampled data using the first two ICs of TICA. (c) The projected coordinates of the first IC of TICA. For every simulation, the last 200 ns (20000 frames) were taken for analysis. Trajectories of the two PH domains were concatenated to make a 40000-frame data and therefore 200000 frames in total. CHARMM36 force-field was used for the first three simulations (sim-1, sim-2 and sim-3) and CHARMM36m was used for the latter two simulations (sim-4 and sim-5).
(TIF)

S1 Movie PTMC calculations of membrane surface absorption of ACAP1 protein by placing the protein at distance from the membrane. A random initial orientation of ACAP1 was used in PTMC simulation, the simulation result indicated that the ACAP1 molecule adsorbed on the membrane surface with the asymmetrical binding.
(MOV)

S2 Movie PTMC calculations of membrane surface absorption of ACAP1 protein by introducing a symmetrical initial condition. We adopt the symmetrical model as the initial orientation in PTMC simulation, still, the asymmetrical binding was found.
(MOV)
