# Supplementary material for: ACAP1 assembles into an unusual protein lattice for membrane deformation through multiple stages
Source: PLoS Comput Biol. 2019 Jul 10;15(7):e1007081. doi: 10.1371/journal.pcbi.1007081 (PMC6663034; doi:10.1371/journal.pcbi.1007081)
Supplement: S1 Table — All residue pairs were summarized from class 1 MDFF trajectory then extracted from those dominating the interaction interface in S3 Fig. The interaction energies were calculated using VMD’s NAMD Energy tool[45]. Number of salt bridges/H-bonds was counted and averaged over the simulation trajectory. Abbreviations: Elec., electrostatic; VDW, Van der Waal’s. (DOCX) [file pcbi.1007081.s001.docx]

**S1 Table. Key contacting residues in the ACAP1^BAR-PH^ protein lattice.** All residue pairs were summarised from class 1 MDFF trajectory then extracted from those dominating the interaction interface in Fig. S3. The interaction energies were calculated using VMD’s NAMD Energy tool^4^. Number of salt bridges/H-bonds was counted and averaged over the simulation trajectory. Abbreviations: Elec., electrostatic; VDW, Van der Waal’s.

|  | **Index** | **Subunits** | **Secondary structure** | **Residues** | **Elec. Energy**  **(kcal/mol)** | **VDW Energy**  **(kcal/mol)** | **Salt bridges** | **Hydrogen bonds** |
| --- | --- | --- | --- | --- | --- | --- | --- | --- |
| **Interface I.**  **Among rows (front)** | **1** | **N** | **α2*** | **99-115** | -21.64 (13.51) | -3.50 (1.82) | - | 0.52 (0.56) |
|  |  | **N-1** | **α2 α3*** | **150-161** |  |  |  |  |
|  | **2** | **N** | **α2*** | **118-126** | -79.50 (23.48) | 2.53 (3.70) | 1.90 (0.00) | 1.80 (0.39) |
|  |  | **N-1** | **α2*** | **118-126** |  |  |  |  |
|  | **3** | **N** | **α2 α3*** | **150-161** | -21.99 (11.52) | -3.86 (2.07) | - | 0.52 (0.56) |
|  |  | **N-1** | **α2*** | **99-115** |  |  |  |  |
|  | **4** | **N** | **α4*** | **236-248** | -51.76 (15.00) | -16.25 (3.48) | - | 1.44 (0.93) |
|  |  | **N-1** | **α4*** | **236-248** |  |  |  |  |
| **Interface II.**  **Same row** | **1** | **N** | **Loop 1**** | **276-282** | -92.51 (34.18) | -19.96 (4.95) | 0.84 (0.08) | 1.36 (0.33) |
|  |  | **N+1** | **α0*** | **1-20** |  |  |  |  |
|  | **2** | **N** | **Loop 1**** | **276-282** | -9.67 (21.29) | -3.17 (1.91) | ~~-~~ | ~~-~~ |
|  |  | **N+1** | **α4*** | **234-245** |  |  |  |  |
| **Interface III.**  **Among rows (back)** | **1** | **N** | **α2*** | **72-85** | -30.26 (21.02) | -0.86 (0.86) | ~~-~~ | ~~-~~ |
|  |  | **N+3** | **Loop 2**** | **304-311** |  |  |  |  |
|  | **2** | **N** | **α2*** | **89-96** | -51.70 (17.78) | -0.68 (2.22) | 0.60 (0.00) | 0.52 (0.00) |
|  |  | **N+2** | **α2*** | **236-240** |  |  |  |  |

* From the BAR domain

** From the PH domain
