# Supplementary material for: ACAP1 assembles into an unusual protein lattice for membrane deformation through multiple stages
Source: PLoS Comput Biol. 2019 Jul 10;15(7):e1007081. doi: 10.1371/journal.pcbi.1007081 (PMC6663034; doi:10.1371/journal.pcbi.1007081)
Supplement: S2 Table — (DOCX) [file pcbi.1007081.s002.docx]

**S2 Table. PTMC simulation parameters and results for BAR domains.**

| Protein | PDB ID | SCD  (C m^-2^) | IS  (M) | Dipole  moment (D) | U_ele_  (kcal mol^-1^) | U_vdw_  (kcal mol^-1^) | U_tot_  (kcal mol^-1^) | cosθ |
| --- | --- | --- | --- | --- | --- | --- | --- | --- |
| ACAP1^BAR-PH^ | 5H3D* | -0.07 | 0.18 | 1236.2 | -41.18 | -16.87 | -58.05 | 0.7 |
|  |  | -0.127 |  |  | -61.95 | -10.49 | -72.44 | -0.9 |
| Endophilin  N-BAR | 1X03 | -0.07 | 0.15 | 1537.1 | -132.91 | -13.55 | -146.46 | -0.9 |
|  |  | -0.127 |  |  | -244.90 | -13.19 | -258.10 | -0.9 |
| F-BAR | 2EFK | -0.07 | 0.15 | 640.3 | -34.94 | -32.36 | -67.30 | -0.4 |
|  |  | -0.127 |  |  | -63.65 | -32.27 | -95.91 | -0.4 |
| I-BAR | 1WDZ | -0.07 | 0.15 | 1004.3 | -144.43 | -29.66 | -174.09 | -0.7 |
|  |  | -0.125 |  |  | -258.50 | -29.64 | -288.14 | -0.7 |

***This publication**
