# Supplementary material for: ACAP1 assembles into an unusual protein lattice for membrane deformation through multiple stages
Source: PLoS Comput Biol. 2019 Jul 10;15(7):e1007081. doi: 10.1371/journal.pcbi.1007081 (PMC6663034; doi:10.1371/journal.pcbi.1007081)
Supplement: S3 Table — (DOCX) [file pcbi.1007081.s003.docx]

**S3 Table. Key** **interacting residues from BAR domains identified during protein recruitment PTMC simulations.**

| Protein | PDB ID | Key interacting residues |
| --- | --- | --- |
| ACAP1^BAR-PH^ | 5H3D | R147, R148, A149, Q150, Q151, F280, K281, D322, E324 |
| Endophilin N-BAR | 1X03 | R174, Q175, G176, K177, I178, E182 |
| F-BAR | 2EFK | K56, E92, K104, Q107, K114, K122, R125, Q160, A167, Q170, K174 |
| I-BAR | 1WDZ | E110, L111, R114, E140, L141, K143, L144, K147 |
