# Supplementary material for: ACAP1 assembles into an unusual protein lattice for membrane deformation through multiple stages
Source: PLoS Comput Biol. 2019 Jul 10;15(7):e1007081. doi: 10.1371/journal.pcbi.1007081 (PMC6663034; doi:10.1371/journal.pcbi.1007081)
Supplement: S4 Table — 500 sequences were used to sample the list of homologues collected from UniProt to the query, among which 152 unique sequences were further used for conservation analysis. The conservation scores were calculated using Bayesian method and presented by conservation level (9—conserved, 1—variable). MSA data is the number of aligned sequences having an amino acid (non-gapped) from the overall number of sequences at each position. Residues that have been mutated in mutagenesis were colored in red. (DOCX) [file pcbi.1007081.s004.docx]

**S4 Table. Statistics of residue conservation using ConSurf (Ashkenazy et al. Nucl. Acids Res. 2016).** 500 sequences were used to sample the list of homologues collected from UniProt to the query, among which 152 unique sequences were further used for conservation analysis. The conservation scores were calculated using Bayesian method and presented by conservation level (9 - conserved, 1 - variable). MSA data is the number of aligned sequences having an amino acid (non-gapped) from the overall number of sequences at each position. Residues that have been mutated in mutagenesis were colored in red.

| Residue:Chain | Conservation level | Score | Confidence interval | | MSA Data |
| --- | --- | --- | --- | --- | --- |
| MET1:A | 9 | -1.193 | -1.506 | -1.086 | 41/152 |
| THR2:A | 5 | 0.025 | -0.403 | 0.397 | 52/152 |
| VAL3:A | 3 | 0.657 | 0.139 | 0.968 | 68/152 |
| LYS4:A | 3 | 0.517 | 0.031 | 0.74 | 87/152 |
| LEU5:A | 6 | -0.384 | -0.674 | -0.161 | 116/152 |
| ASP6:A | 6 | -0.41 | -0.736 | -0.246 | 118/152 |
| PHE7:A | 7 | -0.488 | -0.796 | -0.246 | 119/152 |
| GLU8:A | 4 | 0.309 | -0.069 | 0.552 | 120/152 |
| GLU9:A | 5 | -0.033 | -0.403 | 0.261 | 120/152 |
| CYS10:A | 7 | -0.528 | -0.856 | -0.327 | 120/152 |
| LEU11:A | 4 | 0.44 | 0.031 | 0.74 | 120/152 |
| LYS12:A | 4 | 0.388 | -0.069 | 0.74 | 120/152 |
| ASP13:A | 8 | -1.058 | -1.265 | -0.914 | 120/152 |
| SER14:A | 9 | -1.138 | -1.331 | -1.028 | 120/152 |
| PRO15:A | 6 | -0.438 | -0.736 | -0.246 | 123/152 |
| ARG16:A | 2 | 0.883 | 0.397 | 1.27 | 126/152 |
| PHE17:A | 4 | 0.256 | -0.161 | 0.552 | 129/152 |
| ARG18:A | 9 | -1.174 | -1.406 | -1.086 | 134/152 |
| ALA19:A | 5 | -0.104 | -0.403 | 0.139 | 133/152 |
| SER20:A | 2 | 1.074 | 0.552 | 1.27 | 134/152 |
| ILE21:A | 5 | -0.06 | -0.403 | 0.139 | 133/152 |
| GLU22:A | 7 | -0.573 | -0.856 | -0.403 | 133/152 |
| LEU23:A | 1 | 1.68 | 0.968 | 1.726 | 134/152 |
| VAL24:A | 6 | -0.413 | -0.674 | -0.246 | 134/152 |
| GLU25:A | 9 | -1.225 | -1.406 | -1.144 | 134/152 |
| ALA26:A | 1 | 1.702 | 0.968 | 1.726 | 134/152 |
| GLU27:A | 5 | 0.054 | -0.327 | 0.261 | 134/152 |
| VAL28:A | 7 | -0.737 | -0.971 | -0.61 | 134/152 |
| SER29:A | 3 | 0.761 | 0.261 | 0.968 | 134/152 |
| GLU30:A | 3 | 0.655 | 0.139 | 0.968 | 134/152 |
| LEU31:A | 8 | -0.832 | -1.086 | -0.674 | 134/152 |
| GLU32:A | 9 | -1.301 | -1.506 | -1.203 | 134/152 |
| THR33:A | 5 | 0.078 | -0.246 | 0.261 | 134/152 |
| ARG34:A | 5 | 0.025 | -0.327 | 0.261 | 134/152 |
| LEU35:A | 8 | -0.874 | -1.144 | -0.736 | 134/152 |
| GLU36:A | 4 | 0.354 | -0.069 | 0.552 | 135/152 |
| LYS37:A | 7 | -0.794 | -1.028 | -0.61 | 136/152 |
| LEU38:A | 6 | -0.278 | -0.61 | -0.069 | 137/152 |
| LEU39:A | 8 | -0.919 | -1.144 | -0.796 | 137/152 |
| LYS40:A | 8 | -1.041 | -1.265 | -0.914 | 137/152 |
| LEU41:A | 5 | 0.075 | -0.327 | 0.397 | 137/152 |
| GLY42:A | 6 | -0.468 | -0.796 | -0.246 | 137/152 |
| THR43:A | 4 | 0.163 | -0.161 | 0.397 | 137/152 |
| GLY44:A | 1 | 2.57 | 1.726 | 2.892 | 137/152 |
| LEU45:A | 8 | -0.939 | -1.144 | -0.796 | 138/152 |
| LEU46:A | 6 | -0.395 | -0.674 | -0.161 | 142/152 |
| GLU47:A | 6 | -0.169 | -0.476 | 0.031 | 142/152 |
| SER48:A | 5 | -0.149 | -0.476 | 0.031 | 142/152 |
| GLY49:A | 9 | -1.201 | -1.406 | -1.086 | 142/152 |
| ARG50:A | 6 | -0.37 | -0.674 | -0.161 | 142/152 |
| HIS51:A | 2 | 0.963 | 0.552 | 1.27 | 142/152 |
| TYR52:A | 6 | -0.347 | -0.674 | -0.069 | 143/152 |
| LEU53:A | 1 | 1.437 | 0.74 | 1.726 | 143/152 |
| ALA54:A | 1 | 2.798 | 1.726 | 2.892 | 143/152 |
| ALA55:A | 5 | -0.06 | -0.403 | 0.139 | 143/152 |
| SER56:A | 7 | -0.565 | -0.796 | -0.403 | 143/152 |
| ARG57:A | 5 | -0.065 | -0.403 | 0.139 | 143/152 |
| ALA58:A | 3 | 0.591 | 0.139 | 0.968 | 143/152 |
| PHE59:A | 8 | -0.839 | -1.086 | -0.674 | 143/152 |
| VAL60:A | 3 | 0.598 | 0.139 | 0.968 | 143/152 |
| VAL61:A | 1 | 1.968 | 1.27 | 2.892 | 143/152 |
| GLY62:A | 6 | -0.372 | -0.674 | -0.161 | 143/152 |
| ILE63:A | 5 | 0.052 | -0.327 | 0.261 | 143/152 |
| CYS64:A | 3 | 0.641 | 0.139 | 0.968 | 143/152 |
| ASP65:A | 5 | 0.121 | -0.246 | 0.397 | 143/152 |
| LEU66:A | 7 | -0.792 | -1.086 | -0.61 | 143/152 |
| ALA67:A | 6 | -0.273 | -0.544 | -0.069 | 143/152 |
| ARG68:A | 1 | 2.449 | 1.27 | 2.892 | 143/152 |
| LEU69:A | 1 | 1.208 | 0.74 | 1.726 | 143/152 |
| GLY70:A | 5 | -0.058 | -0.403 | 0.139 | 143/152 |
| PRO71:A | 1 | 2.644 | 1.726 | 2.892 | 141/152 |
| PRO72:A | 1 | 2.167 | 1.27 | 2.892 | 143/152 |
| GLU73:A | 7 | -0.812 | -1.028 | -0.674 | 143/152 |
| PRO74:A | 1 | 2.877 | 1.726 | 2.892 | 143/152 |
| MET75:A | 1 | 1.383 | 0.74 | 1.726 | 143/152 |
| MET76:A | 4 | 0.45 | 0.031 | 0.74 | 143/152 |
| ALA77:A | 1 | 1.418 | 0.74 | 1.726 | 143/152 |
| GLU78:A | 1 | 1.3 | 0.74 | 1.726 | 140/152 |
| CYS79:A | 4 | 0.383 | -0.069 | 0.74 | 140/152 |
| LEU80:A | 8 | -0.996 | -1.265 | -0.856 | 140/152 |
| GLU81:A | 1 | 1.527 | 0.968 | 1.726 | 140/152 |
| LYS82:A | 5 | 0.041 | -0.327 | 0.261 | 140/152 |
| PHE83:A | 7 | -0.644 | -0.914 | -0.476 | 140/152 |
| THR84:A | 5 | -0.002 | -0.327 | 0.261 | 140/152 |
| VAL85:A | 1 | 1.436 | 0.74 | 1.726 | 140/152 |
| SER86:A | 4 | 0.338 | -0.069 | 0.552 | 140/152 |
| LEU87:A | 6 | -0.441 | -0.736 | -0.246 | 141/152 |
| ASN88:A | 5 | 0.16 | -0.161 | 0.397 | 141/152 |
| HIS89:A | 5 | -0.129 | -0.476 | 0.139 | 141/152 |
| LYS90:A | 6 | -0.18 | -0.476 | 0.031 | 141/152 |
| LEU91:A | 2 | 1.003 | 0.552 | 1.27 | 141/152 |
| ASP92:A | 4 | 0.311 | -0.069 | 0.552 | 141/152 |
| SER93:A | 4 | 0.406 | 0.031 | 0.74 | 141/152 |
| HIS94:A | 7 | -0.584 | -0.856 | -0.403 | 142/152 |
| ALA95:A | 2 | 0.828 | 0.397 | 0.968 | 143/152 |
| GLU96:A | 7 | -0.489 | -0.736 | -0.327 | 147/152 |
| LEU97:A | 8 | -0.914 | -1.144 | -0.736 | 146/152 |
| LEU98:A | 6 | -0.271 | -0.61 | -0.069 | 146/152 |
| ASP99:A | 7 | -0.741 | -0.971 | -0.61 | 146/152 |
| ALA100:A | 7 | -0.658 | -0.914 | -0.476 | 147/152 |
| THR101:A | 6 | -0.326 | -0.61 | -0.161 | 148/152 |
| GLN102:A | 7 | -0.733 | -0.971 | -0.61 | 148/152 |
| HIS103:A | 6 | -0.275 | -0.544 | -0.069 | 146/152 |
| THR104:A | 7 | -0.72 | -0.914 | -0.61 | 148/152 |
| LEU105:A | 6 | -0.309 | -0.61 | -0.161 | 148/152 |
| GLN106:A | 1 | 1.252 | 0.74 | 1.726 | 148/152 |
| GLN107:A | 3 | 0.708 | 0.261 | 0.968 | 148/152 |
| GLN108:A | 7 | -0.78 | -1.028 | -0.61 | 148/152 |
| ILE109:A | 7 | -0.633 | -0.914 | -0.476 | 148/152 |
| GLN110:A | 3 | 0.567 | 0.139 | 0.74 | 148/152 |
| THR111:A | 1 | 1.298 | 0.74 | 1.726 | 148/152 |
| LEU112:A | 8 | -1.094 | -1.331 | -0.971 | 148/152 |
| VAL113:A | 4 | 0.303 | -0.069 | 0.552 | 148/152 |
| LYS114:A | 6 | -0.351 | -0.674 | -0.161 | 147/152 |
| GLU115:A | 3 | 0.567 | 0.139 | 0.74 | 146/152 |
| GLY116:A | 6 | -0.232 | -0.544 | -0.069 | 148/152 |
| LEU117:A | 4 | 0.427 | 0.031 | 0.74 | 149/152 |
| ARG118:A | 6 | -0.187 | -0.476 | 0.031 | 149/152 |
| GLY119:A | 3 | 0.769 | 0.261 | 0.968 | 148/152 |
| PHE120:A | 6 | -0.423 | -0.736 | -0.246 | 149/152 |
| ARG121:A | 4 | 0.167 | -0.161 | 0.397 | 150/152 |
| GLU122:A | 4 | 0.33 | -0.069 | 0.552 | 150/152 |
| ALA123:A | 5 | 0.053 | -0.246 | 0.261 | 150/152 |
| ARG124:A | 7 | -0.509 | -0.796 | -0.327 | 151/152 |
| ARG125:A | 6 | -0.204 | -0.544 | 0.031 | 151/152 |
| ASP126:A | 3 | 0.532 | 0.139 | 0.74 | 151/152 |
| PHE127:A | 7 | -0.739 | -1.028 | -0.544 | 151/152 |
| TRP128:A | 4 | 0.281 | -0.069 | 0.552 | 150/152 |
| ARG129:A | 5 | 0.139 | -0.246 | 0.397 | 150/152 |
| GLY130:A | 5 | -0.161 | -0.476 | 0.031 | 151/152 |
| ALA131:A | 8 | -0.871 | -1.086 | -0.736 | 152/152 |
| GLU132:A | 6 | -0.21 | -0.544 | 0.031 | 152/152 |
| SER133:A | 2 | 0.825 | 0.397 | 1.27 | 152/152 |
| LEU134:A | 2 | 1.053 | 0.552 | 1.27 | 152/152 |
| GLU135:A | 6 | -0.346 | -0.61 | -0.161 | 152/152 |
| ALA136:A | 1 | 2.43 | 1.27 | 2.892 | 152/152 |
| ALA137:A | 8 | -0.927 | -1.144 | -0.796 | 152/152 |
| LEU138:A | 6 | -0.197 | -0.544 | 0.031 | 152/152 |
| THR139:A | 1 | 2.848 | 1.726 | 2.892 | 152/152 |
| HIS140:A | 5 | 0.014 | -0.327 | 0.261 | 152/152 |
| ASN141:A | 7 | -0.81 | -1.028 | -0.674 | 152/152 |
| ALA142:A | 8 | -0.928 | -1.144 | -0.796 | 152/152 |
| GLU143:A | 8 | -0.823 | -1.028 | -0.674 | 152/152 |
| VAL144:A | 5 | -0.034 | -0.327 | 0.139 | 152/152 |
| PRO145:A | 2 | 0.852 | 0.397 | 1.27 | 152/152 |
| ARG146:A | 6 | -0.406 | -0.674 | -0.246 | 152/152 |
| ARG147:A | 4 | 0.328 | -0.069 | 0.552 | 151/152 |
| ARG148:A | 7 | -0.512 | -0.796 | -0.327 | 151/152 |
| ALA149:A | 1 | 2.193 | 1.27 | 2.892 | 151/152 |
| GLN150:A | 3 | 0.639 | 0.261 | 0.968 | 151/152 |
| GLU151:A | 7 | -0.643 | -0.914 | -0.476 | 152/152 |
| ALA152:A | 4 | 0.234 | -0.161 | 0.397 | 152/152 |
| GLU153:A | 6 | -0.436 | -0.736 | -0.246 | 152/152 |
| GLU154:A | 6 | -0.483 | -0.736 | -0.327 | 152/152 |
| ALA155:A | 7 | -0.765 | -0.971 | -0.61 | 152/152 |
| GLY156:A | 3 | 0.574 | 0.139 | 0.74 | 152/152 |
| ALA157:A | 3 | 0.554 | 0.139 | 0.74 | 152/152 |
| ALA158:A | 1 | 1.254 | 0.74 | 1.726 | 152/152 |
| LEU159:A | 7 | -0.638 | -0.914 | -0.476 | 152/152 |
| ARG160:A | 1 | 1.421 | 0.74 | 1.726 | 152/152 |
| THR161:A | 4 | 0.304 | -0.069 | 0.552 | 152/152 |
| ALA162:A | 6 | -0.376 | -0.61 | -0.161 | 152/152 |
| ARG163:A | 7 | -0.751 | -0.971 | -0.61 | 152/152 |
| ALA164:A | 5 | -0.147 | -0.476 | 0.031 | 152/152 |
| GLY165:A | 2 | 1.07 | 0.552 | 1.27 | 151/152 |
| TYR166:A | 6 | -0.234 | -0.61 | 0.031 | 152/152 |
| ARG167:A | 5 | -0.052 | -0.403 | 0.139 | 152/152 |
| GLY168:A | 6 | -0.432 | -0.674 | -0.246 | 152/152 |
| ARG169:A | 3 | 0.682 | 0.261 | 0.968 | 152/152 |
| ALA170:A | 7 | -0.591 | -0.856 | -0.403 | 151/152 |
| LEU171:A | 8 | -0.841 | -1.086 | -0.674 | 152/152 |
| ASP172:A | 9 | -1.279 | -1.506 | -1.203 | 152/152 |
| TYR173:A | 7 | -0.781 | -1.028 | -0.61 | 152/152 |
| ALA174:A | 8 | -0.866 | -1.086 | -0.736 | 152/152 |
| LEU175:A | 6 | -0.485 | -0.796 | -0.246 | 152/152 |
| GLN176:A | 8 | -0.86 | -1.086 | -0.736 | 152/152 |
| ILE177:A | 6 | -0.462 | -0.736 | -0.327 | 151/152 |
| ASN178:A | 8 | -0.927 | -1.144 | -0.796 | 151/152 |
| VAL179:A | 5 | 0.038 | -0.327 | 0.261 | 151/152 |
| ILE180:A | 5 | 0.001 | -0.327 | 0.261 | 151/152 |
| GLU181:A | 6 | -0.34 | -0.61 | -0.161 | 151/152 |
| ASP182:A | 5 | -0.009 | -0.327 | 0.139 | 151/152 |
| LYS183:A | 5 | -0.125 | -0.476 | 0.139 | 151/152 |
| ARG184:A | 7 | -0.53 | -0.796 | -0.327 | 151/152 |
| LYS185:A | 6 | -0.288 | -0.61 | -0.069 | 151/152 |
| PHE186:A | 4 | 0.483 | 0.031 | 0.74 | 151/152 |
| ASP187:A | 6 | -0.279 | -0.544 | -0.069 | 151/152 |
| ILE188:A | 5 | -0.092 | -0.403 | 0.139 | 150/152 |
| MET189:A | 7 | -0.641 | -0.914 | -0.476 | 150/152 |
| GLU190:A | 3 | 0.79 | 0.397 | 0.968 | 150/152 |
| PHE191:A | 6 | -0.373 | -0.61 | -0.161 | 151/152 |
| VAL192:A | 4 | 0.343 | -0.069 | 0.552 | 151/152 |
| LEU193:A | 7 | -0.71 | -0.971 | -0.544 | 151/152 |
| ARG194:A | 8 | -0.855 | -1.086 | -0.736 | 151/152 |
| LEU195:A | 4 | 0.232 | -0.161 | 0.552 | 151/152 |
| VAL196:A | 7 | -0.644 | -0.856 | -0.476 | 150/152 |
| GLU197:A | 2 | 0.837 | 0.397 | 1.27 | 151/152 |
| ALA198:A | 8 | -1.043 | -1.203 | -0.914 | 151/152 |
| GLN199:A | 7 | -0.614 | -0.856 | -0.476 | 150/152 |
| ALA200:A | 2 | 1 | 0.552 | 1.27 | 150/152 |
| THR201:A | 4 | 0.201 | -0.161 | 0.397 | 150/152 |
| HIS202:A | 5 | -0.04 | -0.403 | 0.261 | 150/152 |
| PHE203:A | 6 | -0.466 | -0.796 | -0.246 | 151/152 |
| GLN204:A | 6 | -0.388 | -0.674 | -0.246 | 151/152 |
| GLN205:A | 7 | -0.808 | -1.028 | -0.674 | 151/152 |
| GLY206:A | 8 | -0.935 | -1.203 | -0.796 | 150/152 |
| HIS207:A | 2 | 1.025 | 0.552 | 1.27 | 152/152 |
| GLU208:A | 5 | 0.038 | -0.327 | 0.261 | 152/152 |
| GLU209:A | 7 | -0.669 | -0.914 | -0.476 | 152/152 |
| LEU210:A | 5 | -0.029 | -0.403 | 0.261 | 152/152 |
| SER211:A | 1 | 1.173 | 0.74 | 1.726 | 150/152 |
| ARG212:A | 5 | 0.091 | -0.246 | 0.261 | 152/152 |
| LEU213:A | 4 | 0.214 | -0.161 | 0.552 | 152/152 |
| SER214:A | 1 | 1.707 | 0.968 | 1.726 | 152/152 |
| GLN215:A | 2 | 0.963 | 0.397 | 1.27 | 152/152 |
| TYR216:A | 4 | 0.366 | -0.069 | 0.74 | 152/152 |
| ARG217:A | 7 | -0.492 | -0.736 | -0.327 | 152/152 |
| LYS218:A | 5 | -0.028 | -0.327 | 0.139 | 152/152 |
| GLU219:A | 1 | 2.81 | 1.726 | 2.892 | 152/152 |
| LEU220:A | 7 | -0.641 | -0.914 | -0.476 | 151/152 |
| GLY221:A | 5 | 0.029 | -0.327 | 0.261 | 152/152 |
| ALA222:A | 1 | 1.83 | 0.968 | 1.726 | 152/152 |
| GLN223:A | 6 | -0.414 | -0.674 | -0.246 | 152/152 |
| LEU224:A | 5 | -0.109 | -0.476 | 0.139 | 150/152 |
| HIS225:A | 3 | 0.694 | 0.261 | 0.968 | 150/152 |
| GLN226:A | 2 | 1.132 | 0.552 | 1.27 | 150/152 |
| LEU227:A | 5 | -0.093 | -0.403 | 0.139 | 150/152 |
| VAL228:A | 6 | -0.264 | -0.544 | -0.069 | 150/152 |
| LEU229:A | 2 | 0.938 | 0.397 | 1.27 | 150/152 |
| ASN230:A | 4 | 0.464 | 0.031 | 0.74 | 150/152 |
| SER231:A | 5 | -0.026 | -0.327 | 0.139 | 150/152 |
| ALA232:A | 4 | 0.322 | -0.069 | 0.552 | 150/152 |
| ARG233:A | 4 | 0.484 | 0.031 | 0.74 | 150/152 |
| GLU234:A | 5 | 0.027 | -0.327 | 0.261 | 150/152 |
| LYS235:A | 5 | -0.137 | -0.476 | 0.031 | 151/152 |
| ARG236:A | 8 | -1.049 | -1.265 | -0.914 | 151/152 |
| ASP237:A | 3 | 0.584 | 0.139 | 0.968 | 151/152 |
| MET238:A | 8 | -0.913 | -1.144 | -0.796 | 151/152 |
| GLU239:A | 8 | -1.012 | -1.203 | -0.914 | 151/152 |
| GLN240:A | 4 | 0.211 | -0.161 | 0.397 | 151/152 |
| ARG241:A | 6 | -0.348 | -0.61 | -0.161 | 151/152 |
| HIS242:A | 8 | -1.118 | -1.331 | -1.028 | 151/152 |
| VAL243:A | 5 | 0.13 | -0.161 | 0.397 | 151/152 |
| LEU244:A | 1 | 1.29 | 0.74 | 1.726 | 149/152 |
| LEU245:A | 6 | -0.408 | -0.674 | -0.246 | 150/152 |
| LYS246:A | 7 | -0.716 | -0.971 | -0.544 | 150/152 |
| GLN247:A | 5 | 0.01 | -0.327 | 0.261 | 149/152 |
| LYS248:A | 5 | -0.051 | -0.403 | 0.139 | 147/152 |
| GLU249:A | 3 | 0.513 | 0.139 | 0.74 | 145/152 |
| LEU250:A | 1 | 2.651 | 1.726 | 2.892 | 145/152 |
| GLY251:A | 4 | 0.365 | 0.031 | 0.552 | 145/152 |
| GLY252:A | 1 | 2.851 | 1.726 | 2.892 | 147/152 |
| GLU253:A | 4 | 0.234 | -0.161 | 0.552 | 145/152 |
| GLU254:A | 1 | 2.664 | 1.726 | 2.892 | 145/152 |
| PRO255:A | 1 | 2.143 | 1.27 | 2.892 | 115/152 |
| GLU256:A | 1 | 1.426 | 0.74 | 1.726 | 115/152 |
| PRO257:A | 1 | 2.818 | 1.726 | 2.892 | 116/152 |
| SER258:A | 3 | 0.741 | 0.261 | 0.968 | 116/152 |
| LEU259:A | 1 | 2.586 | 1.726 | 2.892 | 138/152 |
| ARG260:A | 1 | 1.655 | 0.968 | 1.726 | 138/152 |
| GLU261:A | 1 | 1.316 | 0.74 | 1.726 | 143/152 |
| GLY262:A | 1 | 1.376 | 0.74 | 1.726 | 147/152 |
| PRO263:A | 1 | 2.795 | 1.726 | 2.892 | 147/152 |
| GLY264:A | 1 | 1.139 | 0.552 | 1.27 | 147/152 |
| GLY265:A | 1 | 1.686 | 0.968 | 1.726 | 147/152 |
| LEU266:A | 3 | 0.635 | 0.261 | 0.968 | 147/152 |
| VAL267:A | 1 | 1.327 | 0.74 | 1.726 | 147/152 |
| MET268:A | 7 | -0.568 | -0.796 | -0.403 | 147/152 |
| GLU269:A | 7 | -0.593 | -0.856 | -0.403 | 148/152 |
| GLY270:A | 8 | -0.988 | -1.265 | -0.856 | 148/152 |
| HIS271:A | 6 | -0.294 | -0.61 | -0.069 | 148/152 |
| LEU272:A | 8 | -1.126 | -1.331 | -1.028 | 148/152 |
| PHE273:A | 6 | -0.369 | -0.674 | -0.161 | 148/152 |
| LYS274:A | 9 | -1.146 | -1.331 | -1.028 | 148/152 |
| ARG275:A | 8 | -0.949 | -1.144 | -0.796 | 148/152 |
| ALA276:A | 7 | -0.779 | -0.971 | -0.61 | 148/152 |
| SER277:A | 8 | -1.025 | -1.203 | -0.914 | 148/152 |
| ASN278:A | 8 | -0.908 | -1.144 | -0.796 | 147/152 |
| ALA279:A | 8 | -1.055 | -1.265 | -0.971 | 147/152 |
| PHE280:A | 5 | -0.134 | -0.476 | 0.139 | 147/152 |
| LYS281:A | 9 | -1.142 | -1.331 | -1.028 | 147/152 |
| THR282:A | 8 | -0.876 | -1.086 | -0.736 | 147/152 |
| TRP283:A | 6 | -0.477 | -0.856 | -0.246 | 147/152 |
| SER284:A | 7 | -0.605 | -0.856 | -0.476 | 148/152 |
| ARG285:A | 9 | -1.351 | -1.603 | -1.265 | 148/152 |
| ARG286:A | 8 | -1.115 | -1.331 | -1.028 | 148/152 |
| TRP287:A | 8 | -0.922 | -1.265 | -0.736 | 147/152 |
| PHE288:A | 9 | -1.32 | -1.603 | -1.203 | 147/152 |
| THR289:A | 6 | -0.449 | -0.674 | -0.246 | 147/152 |
| ILE290:A | 8 | -1.017 | -1.203 | -0.914 | 147/152 |
| GLN291:A | 6 | -0.339 | -0.61 | -0.161 | 147/152 |
| SER292:A | 7 | -0.538 | -0.796 | -0.403 | 147/152 |
| ASN293:A | 8 | -0.875 | -1.086 | -0.736 | 147/152 |
| GLN294:A | 8 | -0.855 | -1.086 | -0.736 | 147/152 |
| LEU295:A | 9 | -1.33 | -1.603 | -1.265 | 147/152 |
| VAL296:A | 7 | -0.772 | -0.971 | -0.61 | 148/152 |
| TYR297:A | 9 | -1.186 | -1.406 | -1.086 | 148/152 |
| GLN298:A | 7 | -0.587 | -0.856 | -0.403 | 147/152 |
| LYS299:A | 9 | -1.231 | -1.406 | -1.144 | 146/152 |
| LYS300:A | 7 | -0.689 | -0.914 | -0.544 | 147/152 |
| TYR301:A | 2 | 1.123 | 0.552 | 1.27 | 144/152 |
| LYS302:A | 5 | -0.124 | -0.476 | 0.139 | 144/152 |
| ASP303:A | 5 | -0.071 | -0.403 | 0.139 | 147/152 |
| PRO304:A | 1 | 2.775 | 1.726 | 2.892 | 147/152 |
| VAL305:A | 1 | 2.281 | 1.27 | 2.892 | 145/152 |
| THR306:A | 8 | -0.939 | -1.144 | -0.796 | 145/152 |
| VAL307:A | 8 | -0.89 | -1.086 | -0.736 | 145/152 |
| VAL308:A | 9 | -1.234 | -1.406 | -1.144 | 145/152 |
| VAL309:A | 8 | -1.088 | -1.265 | -0.971 | 145/152 |
| ASP310:A | 5 | -0.155 | -0.476 | 0.031 | 145/152 |
| ASP311:A | 9 | -1.462 | -1.603 | -1.406 | 145/152 |
| LEU312:A | 9 | -1.332 | -1.603 | -1.265 | 145/152 |
| ARG313:A | 9 | -1.415 | -1.603 | -1.331 | 145/152 |
| LEU314:A | 9 | -1.328 | -1.603 | -1.265 | 145/152 |
| CYS315:A | 9 | -1.389 | -1.603 | -1.331 | 145/152 |
| THR316:A | 8 | -0.856 | -1.086 | -0.736 | 145/152 |
| VAL317:A | 9 | -1.443 | -1.603 | -1.406 | 145/152 |
| LYS318:A | 8 | -0.925 | -1.144 | -0.796 | 145/152 |
| LEU319:A | 2 | 0.821 | 0.397 | 1.27 | 145/152 |
| CYS320:A | 3 | 0.571 | 0.139 | 0.968 | 145/152 |
| PRO321:A | 1 | 1.322 | 0.74 | 1.726 | 144/152 |
| ASP322:A | 5 | -0.151 | -0.476 | 0.031 | 145/152 |
| SER323:A | 2 | 1.001 | 0.552 | 1.27 | 145/152 |
| GLU324:A | 6 | -0.342 | -0.61 | -0.161 | 145/152 |
| ARG325:A | 9 | -1.461 | -1.603 | -1.406 | 145/152 |
| ARG326:A | 9 | -1.411 | -1.603 | -1.331 | 145/152 |
| PHE327:A | 9 | -1.185 | -1.406 | -1.086 | 145/152 |
| CYS328:A | 9 | -1.168 | -1.406 | -1.028 | 145/152 |
| PHE329:A | 9 | -1.318 | -1.506 | -1.203 | 145/152 |
| GLU330:A | 9 | -1.249 | -1.506 | -1.144 | 145/152 |
| VAL331:A | 9 | -1.248 | -1.406 | -1.144 | 145/152 |
| VAL332:A | 8 | -0.947 | -1.144 | -0.796 | 145/152 |
| SER333:A | 9 | -1.417 | -1.603 | -1.331 | 145/152 |
| THR334:A | 8 | -0.993 | -1.203 | -0.856 | 146/152 |
| SER335:A | 5 | 0.117 | -0.246 | 0.397 | 146/152 |
| LYS336:A | 8 | -1.08 | -1.265 | -0.971 | 144/152 |
| SER337:A | 8 | -1.061 | -1.265 | -0.971 | 144/152 |
| CYS338:A | 8 | -0.942 | -1.203 | -0.796 | 144/152 |
| LEU339:A | 3 | 0.719 | 0.261 | 0.968 | 144/152 |
| LEU340:A | 8 | -0.826 | -1.086 | -0.674 | 144/152 |
| GLN341:A | 9 | -1.418 | -1.603 | -1.331 | 144/152 |
| ALA342:A | 9 | -1.394 | -1.603 | -1.331 | 144/152 |
| ASP343:A | 7 | -0.772 | -1.028 | -0.61 | 145/152 |
| SER344:A | 9 | -1.259 | -1.406 | -1.203 | 145/152 |
| GLU345:A | 6 | -0.223 | -0.544 | 0.031 | 145/152 |
| ARG346:A | 4 | 0.436 | 0.031 | 0.74 | 145/152 |
| LEU347:A | 3 | 0.623 | 0.139 | 0.968 | 144/152 |
| LEU348:A | 5 | -0.16 | -0.476 | 0.031 | 144/152 |
| GLN349:A | 4 | 0.43 | 0.031 | 0.74 | 142/152 |
| LEU350:A | 4 | 0.266 | -0.069 | 0.552 | 143/152 |
| TRP351:A | 8 | -1.085 | -1.406 | -0.914 | 143/152 |
| VAL352:A | 6 | -0.269 | -0.544 | -0.069 | 143/152 |
| SER353:A | 2 | 1.055 | 0.552 | 1.27 | 143/152 |
| ALA354:A | 8 | -1.013 | -1.203 | -0.914 | 143/152 |
| VAL355:A | 7 | -0.626 | -0.856 | -0.476 | 144/152 |
| GLN356:A | 8 | -1.037 | -1.265 | -0.914 | 144/152 |
| SER357:A | 3 | 0.514 | 0.139 | 0.74 | 144/152 |
| SER358:A | 8 | -0.876 | -1.086 | -0.736 | 144/152 |
| ILE359:A | 9 | -1.332 | -1.506 | -1.265 | 143/152 |
| ALA360:A | 6 | -0.326 | -0.61 | -0.161 | 144/152 |
| SER361:A | 5 | 0.029 | -0.327 | 0.261 | 144/152 |
| ALA362:A | 9 | -1.328 | -1.506 | -1.265 | 144/152 |
| PHE363:A | 6 | -0.244 | -0.61 | 0.031 | 143/152 |
| SER364:A | 6 | -0.24 | -0.544 | -0.069 | 143/152 |
