# Supplementary figures and images for: ACAP1 assembles into an unusual protein lattice for membrane deformation through multiple stages
Source: PLoS Comput Biol. 2019 Jul 10;15(7):e1007081. doi: 10.1371/journal.pcbi.1007081 (PMC6663034; doi:10.1371/journal.pcbi.1007081)

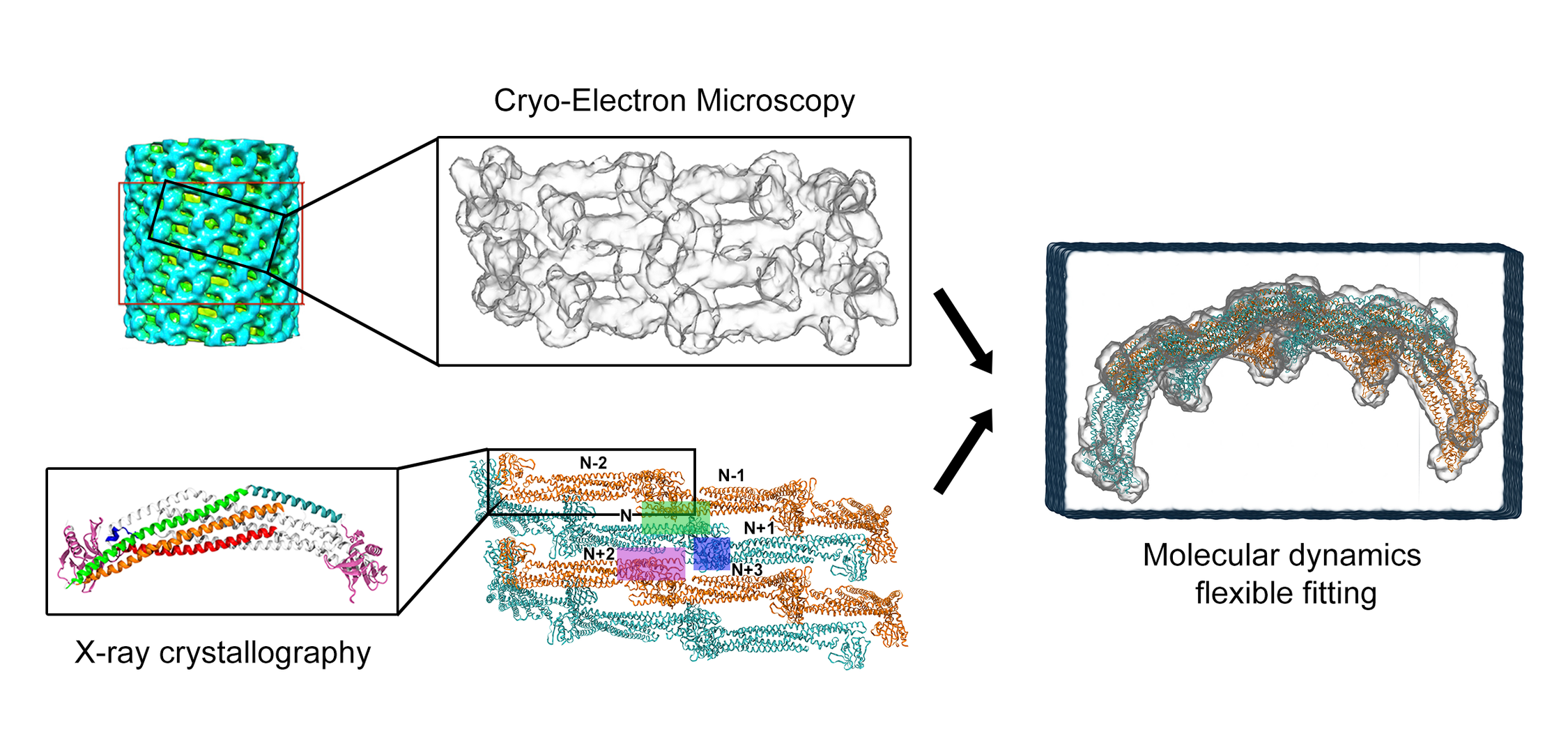

Supplement: S1 Fig — Surface diagram of the ACAP1BAR-PH protein lattice in the class 1 MDFF simulation with dimers labelled. Three interaction interfaces are enclosed with three boxes of colours: green for the front interactions (Interface I), blue for same-row interactions (Interface II), and magenta for the back interactions (Interface III). (TIF) [file pcbi.1007081.s005.tif]

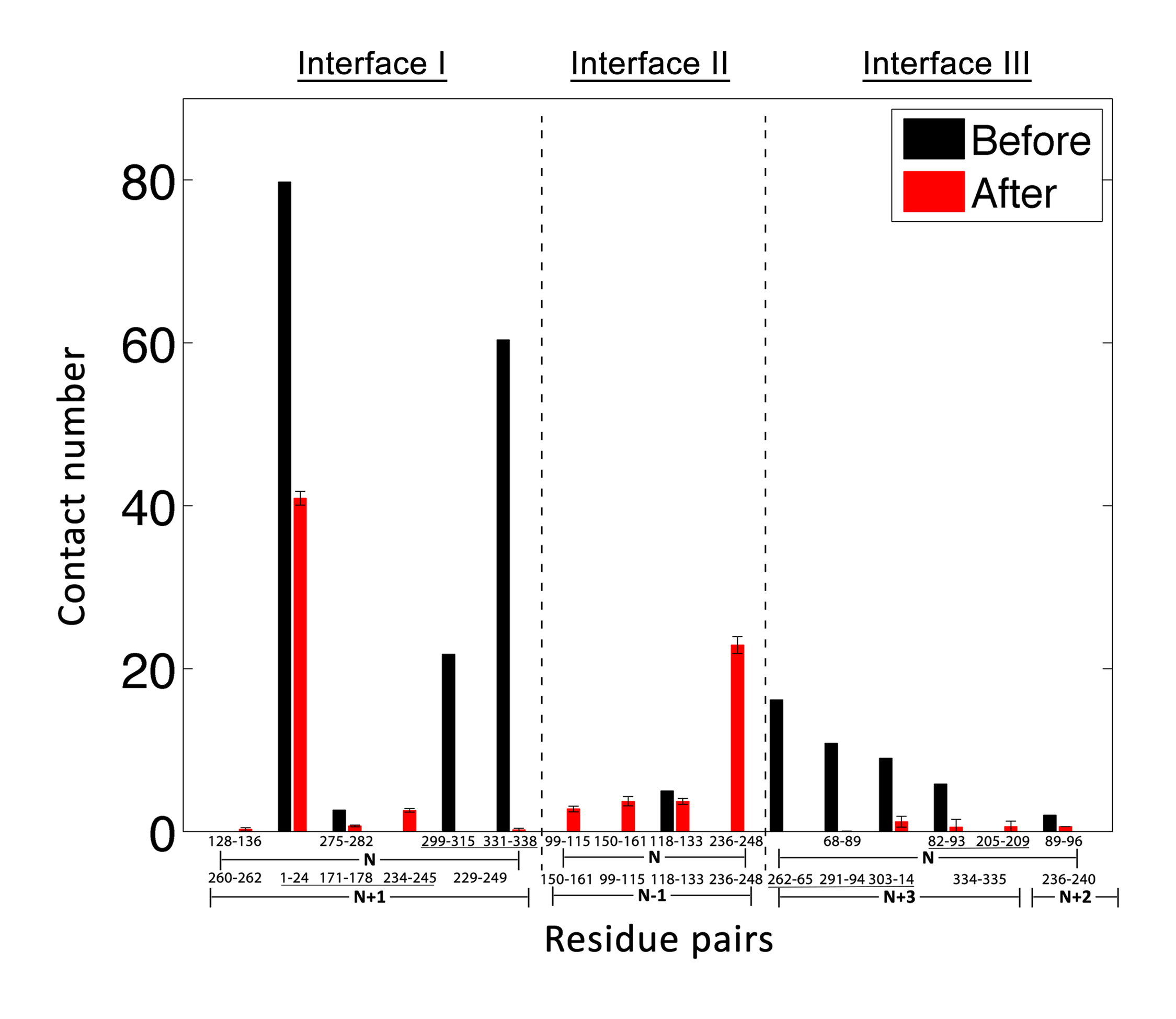

Supplement: S2 Fig — Residue pairs before and after the MDFF refinement were colored in black and red respectively. (TIF) [file pcbi.1007081.s006.tif]

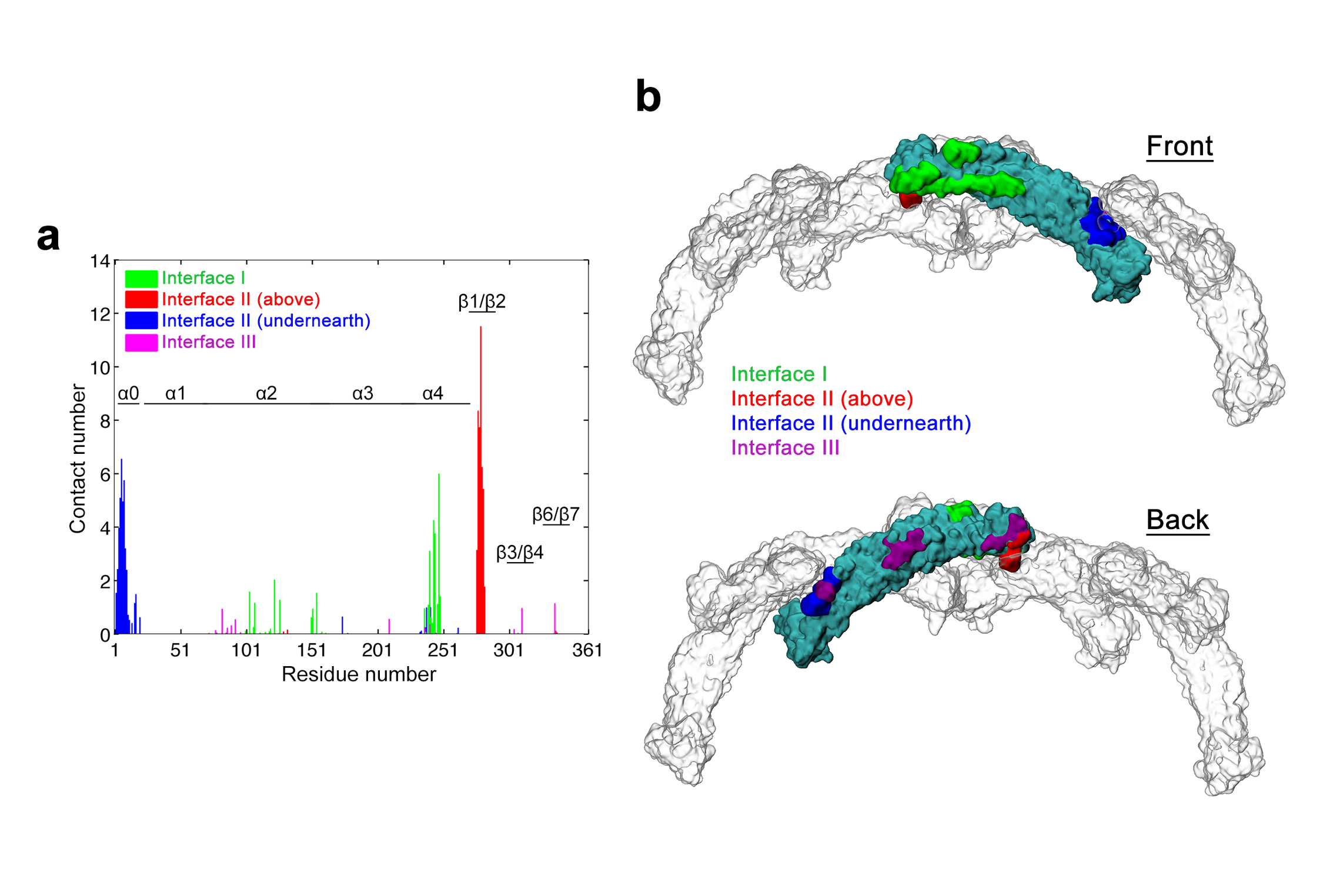

Supplement: S3 Fig — (a) Contact numbers for each residue coloured according to different interacting regions for class 1. Different colours stand for different interacting interfaces, which are consistent with colours shown in Fig 1. An additional colour (red) has been introduced to Interface II and was used to denote residues from the dimer geometrically above the adjacent dimer at the interface. (b) Residue contributions in a have been projected onto the molecular surface of an ACAP1BAR-PH dimer. The adjacent dimers have been shown in transparent. (TIF) [file pcbi.1007081.s007.tif]

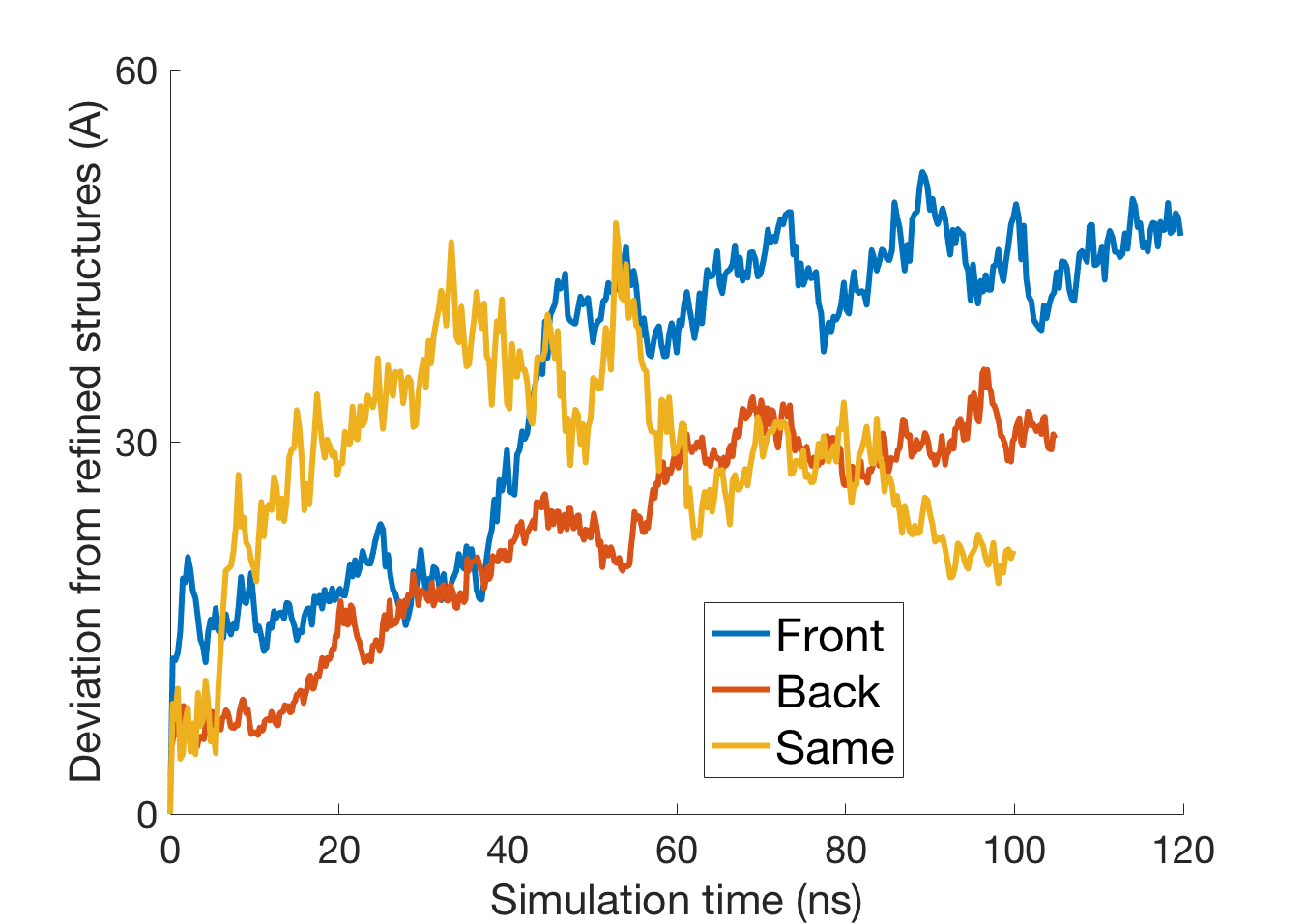

Supplement: S4 Fig — To compute the root-mean-square deviation (RMSD) of the mutated protein dimer with respect to its refined structure, one of the dimers from an equilibrated mutated tetramer MD trajectory was aligned to its refined structure obtained from the MDFF simulation, which also served as the initial structure for the mutated tetramer MD simulations. Three independent simulations were performed with mutations stated in experiments and shown in different colors. (TIF) [file pcbi.1007081.s008.tif]

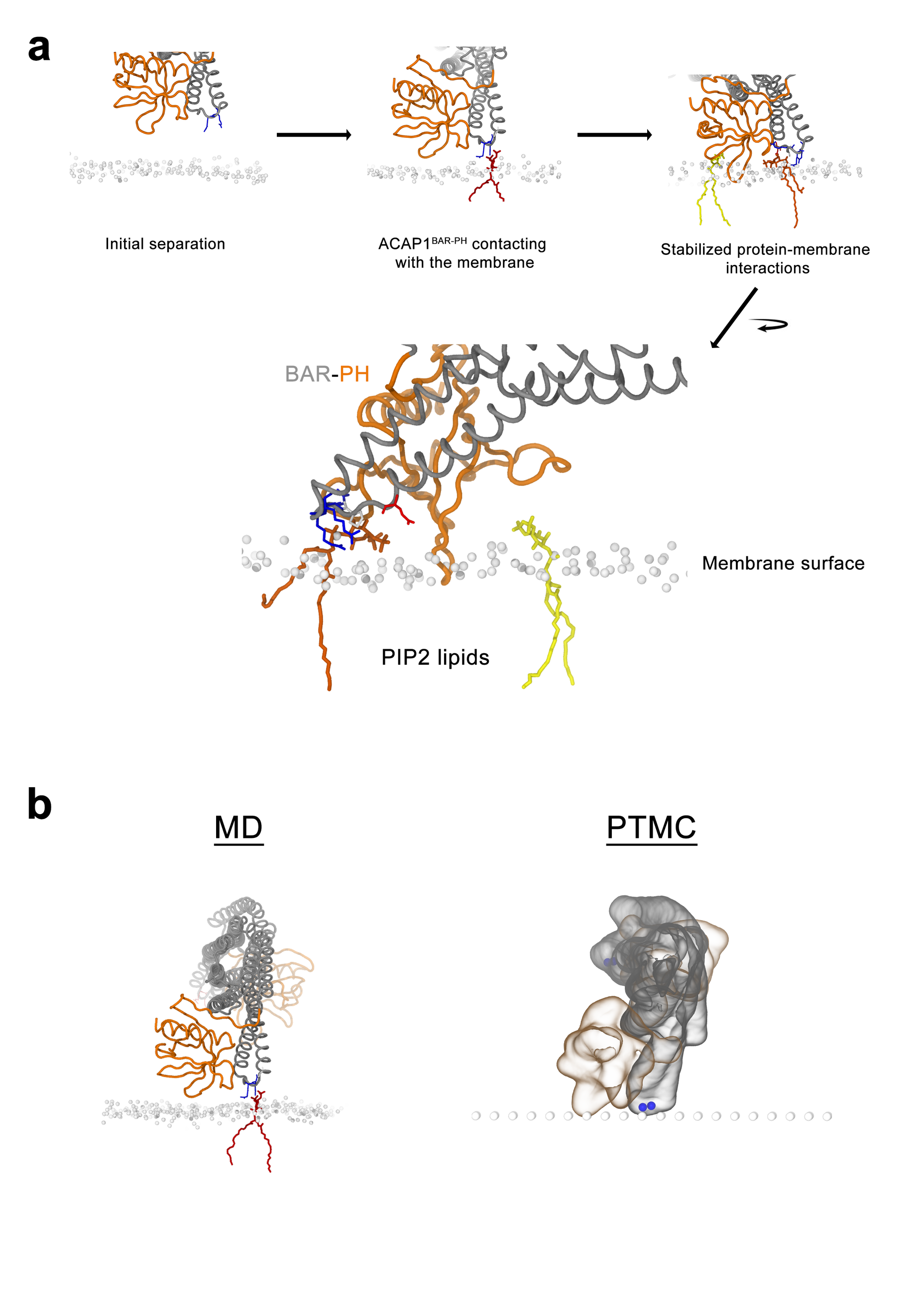

Supplement: S5 Fig — (a) Residues on the BAR domain (especially R147 and R148) were highlighted during the recruitment process. Residues R147 and R148 locate at the distal ends of the BAR domain (initially colored in blue). Residues from the BAR domain that were within 4A of a PIP2 molecule will be shown explicitly and colored according to their electrostatic properties (Blue for positive, red for negative, green to polar and white for hydrophobic). PIP2 molecules that were within 8A of the dimer will also be shown explicitly. (b) A slight “lying-down” orientation was observed for the BAR domain in both MD and PTMC simulations. Residues R147 and R148 were shown in blue spheres in the PTMC snapshot, showing a very similar way of contact seen in the MD snapshots. (TIF) [file pcbi.1007081.s009.tif]

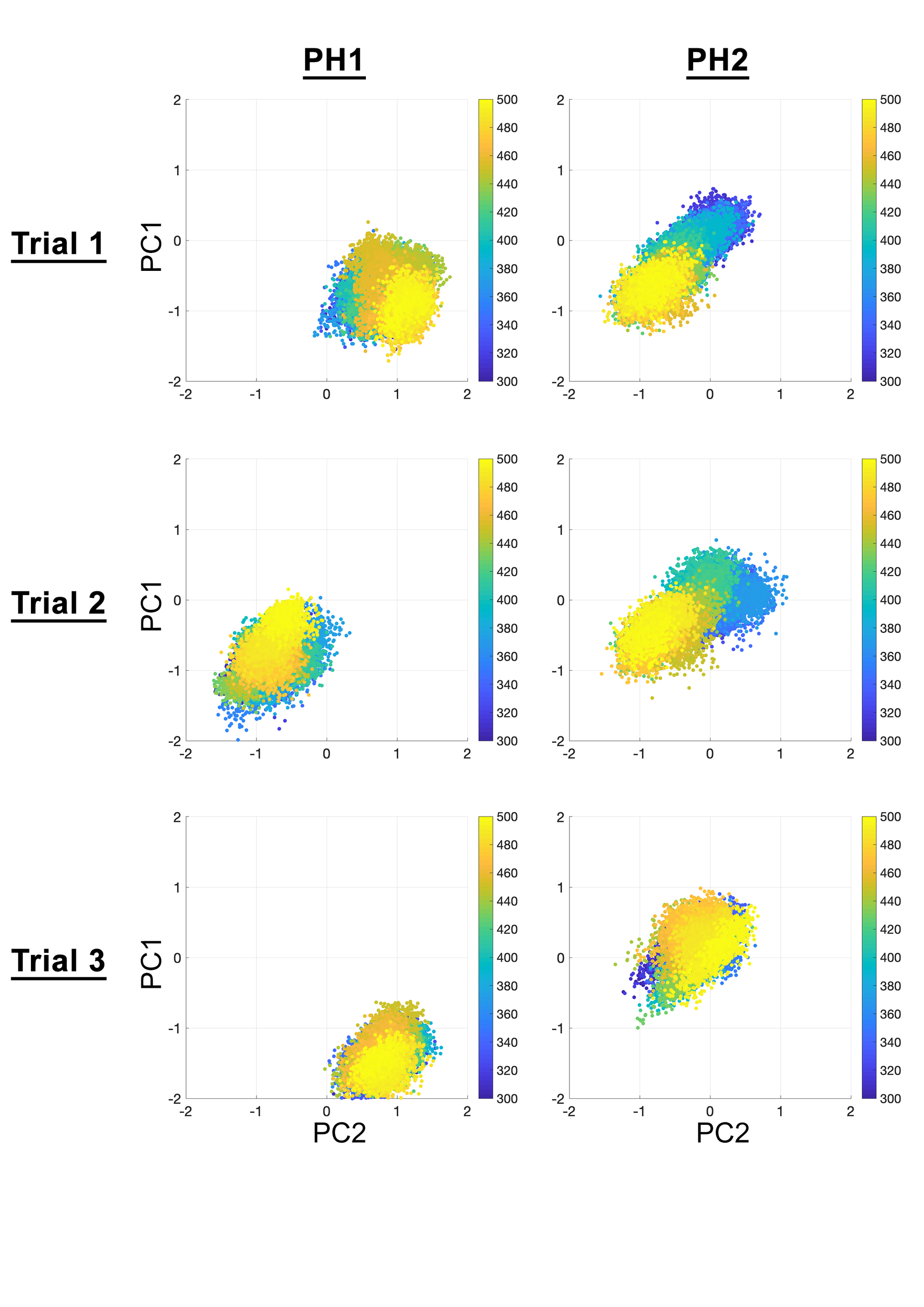

Supplement: S6 Fig — The whole 500 ns trajectories were used to compute the principle components. Last 200 ns of the trajectories were projected onto the first two principle components. Colorbar represents the simulation time. (TIF) [file pcbi.1007081.s010.tif]

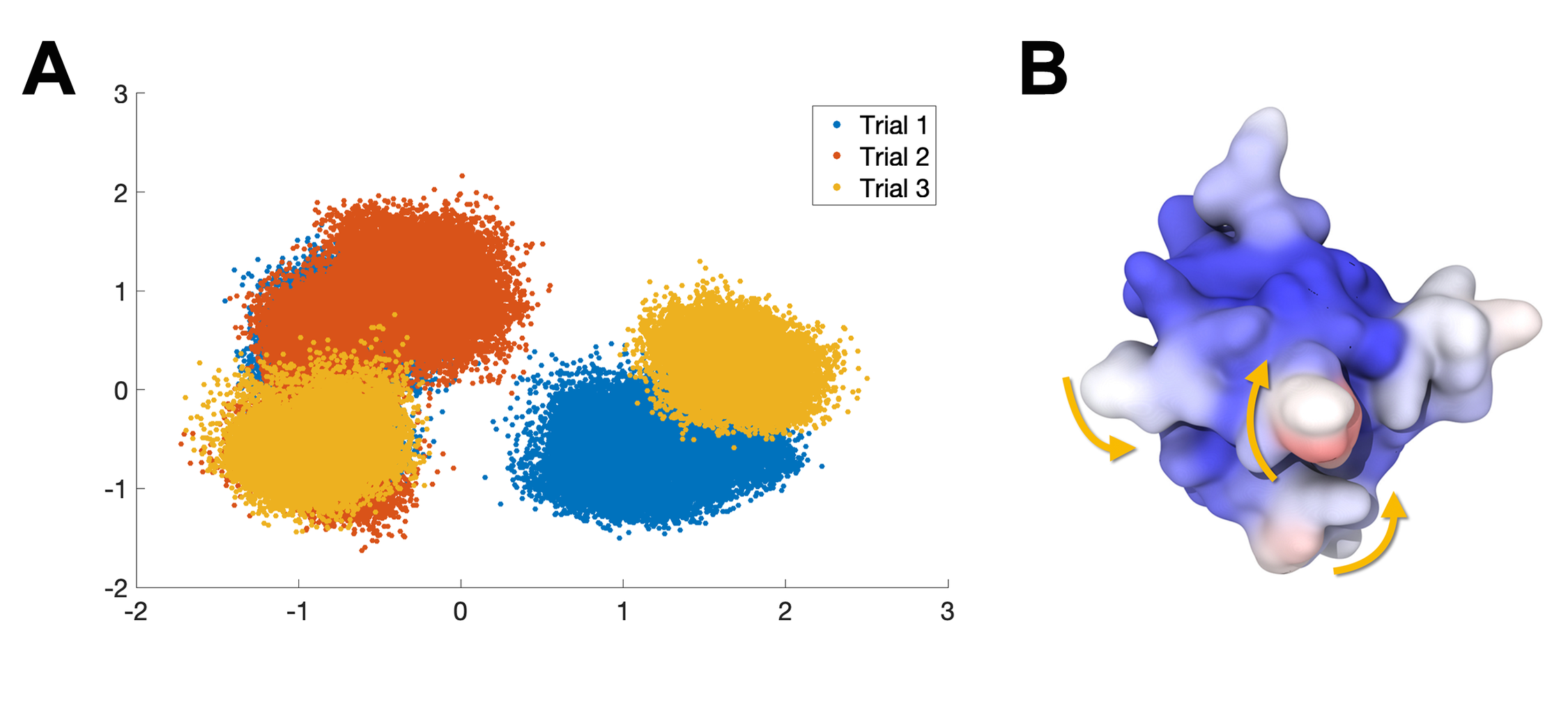

Supplement: S7 Fig — (a) Three independent MD trajectories were projected onto the first two principle components. Trial 1 and 3 have been clearly identified to have two forms of motion along the first principle of component of dynamics in fluctuations (PC1) indicating that the two PH domains underwent different dynamics. Trial 2 was not observed to have such distinct dynamics along PC1, indicating the possibility of interchangeable of the two dynamical states. (b) For the sake of clarity, the (PC1) was represented using arrows on the molecular surface of PH domain shown in Fig 5b. This PC1 has shown that motions in residues of Loop 1, Loop 3 and Loop 4 have contributed to most of the residual fluctuation and distinguish the two dynamics of the PH domain which is in consistent with the RMSF profile in Fig 5c. (TIF) [file pcbi.1007081.s011.tif]

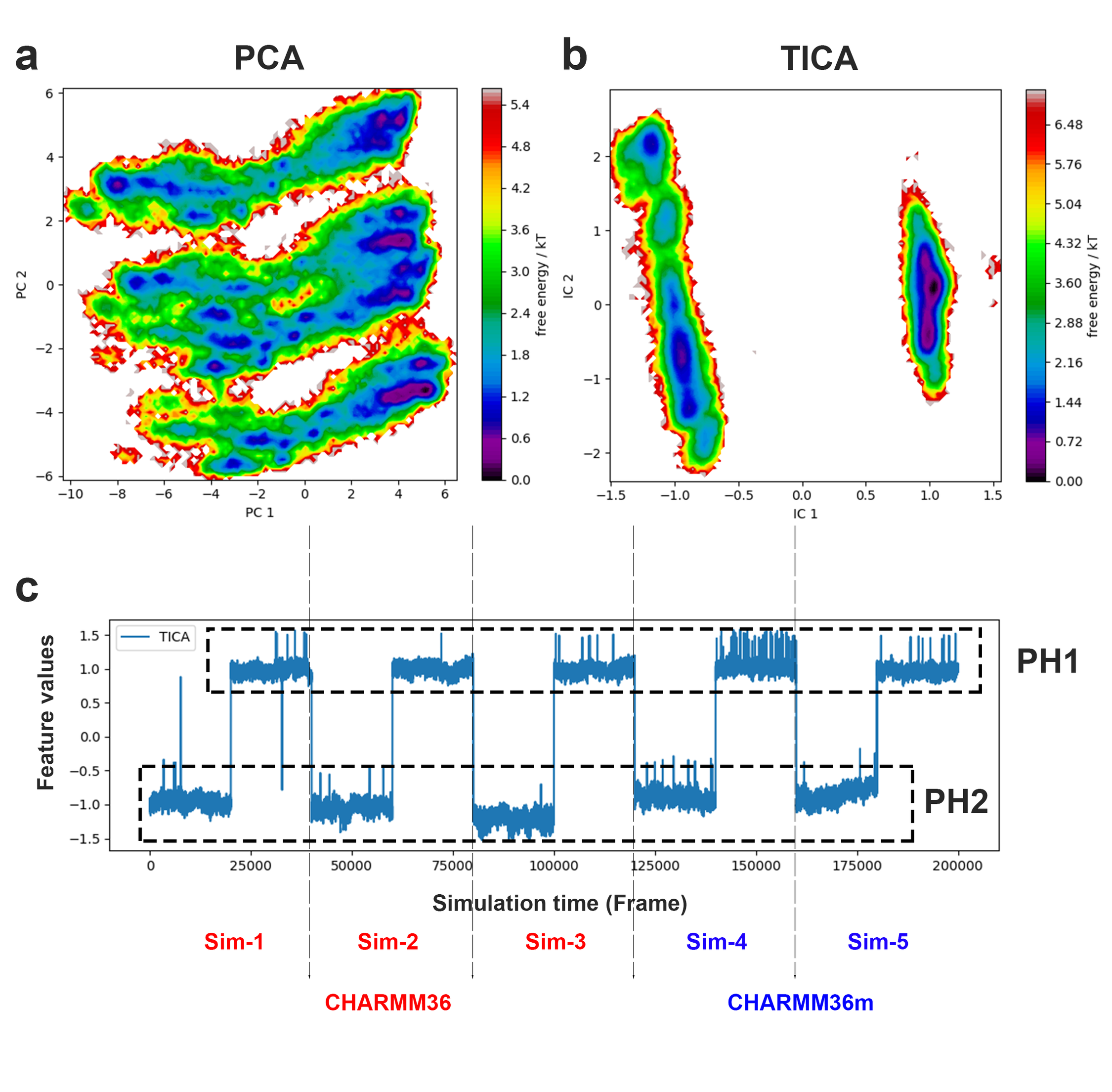

Supplement: S8 Fig — (a) A pseudo free energy surface calculated from the sample densities using the first two PCs of PCA. (b) A pseudo free energy surface of the sampled data using the first two ICs of TICA. (c) The projected coordinates of the first IC of TICA. For every simulation, the last 200 ns (20000 frames) were taken for analysis. Trajectories of the two PH domains were concatenated to make a 40000-frame data and therefore 200000 frames in total. CHARMM36 force-field was used for the first three simulations (sim-1, sim-2 and sim-3) and CHARMM36m was used for the latter two simulations (sim-4 and sim-5). (TIF) [file pcbi.1007081.s012.tif]
